# Multi-omics insights into key microorganisms and metabolites in Tibetan sheep’s high-altitude adaptation

**DOI:** 10.3389/fmicb.2025.1616555

**Published:** 2025-06-18

**Authors:** Jing Wang, Jianbin Liu, Tingting Guo, Chen Zheng, Fan Wang, Ting Liu, Chao Yuan, Zengkui Lu

**Affiliations:** ^1^Key Laboratory of Animal Genetics and Breeding on Tibetan Plateau, Ministry of Agriculture and Rural Affairs, Lanzhou Institute of Husbandry and Pharmaceutical Sciences, Chinese Academy of Agricultural Sciences, Lanzhou, China; ^2^Sheep Breeding Engineering Technology Research Center of Chinese Academy of Agricultural Sciences, Lanzhou, China; ^3^College of Animal Science and Technology, Gansu Agricultural University, Lanzhou, China; ^4^College of Life Science and Engineering, Northwest Minzu University, Lanzhou, China

**Keywords:** Tibetan sheep, altitudes, microorganisms, metabolites, adaptability

## Abstract

Tibetan sheep gastrointestinal microbial communities and metabolites showed adaptive differences with altitude, but we do not know which flora or metabolites may play an important role in acclimatization to the altitude environment. Therefore, we systematically analyzed the microbial structure and metabolites in the rumen and feces of Tibetan sheep at two altitudes (4,424 m and 2,364 m) using amplicon sequencing and untargeted metabolomics. The results showed that the bacterial communities differed greatly between the two groups, with high altitude Tibetan sheep having a higher forage fermentation capacity, and the abundance of some bacteria and fungi that were conducive to the decomposition of cellulose in rumen fluid increased significantly (especially Bacteroidota, Neocallimastigomycota, and Ascomycota), and the short chain fatty acids and NH_3_-N produced by metabolism also increased. There was also a significant increase in the abundance of Naganishia, which is prone to survive in extreme environments. In addition, the metabolite profiles in the rumen and feces of Tibetan sheep at two altitudes were also significantly different, and further correlation analysis showed that the differential bacteria in the rumen were mainly related to the products related to amino acid metabolism and lipid metabolism, and the differential bacteria in the feces were mainly correlated with some metabolites related to antibacterial, anti-inflammatory, anti-tumor and other disease treatment components. Collectively, these changes in microbiota and metabolites may have facilitated the adaptation of Tibetan sheep to the harsh plateau environment, contributing to their better survival and reproduction. This study provides a basis for research on the mechanisms of adaptation of Tibetan sheep to the plateau environment.

## 1 Introduction

Tibetan sheep are the dominant livestock species and important genetic resources in the Tibetan Plateau. They are not only the main source of meat, hair and economic resources for local herdsmen (Xu et al., 2017), but also playing a key role in securing livelihoods for plateau communities. They usually live in the Tibetan Plateau at an altitude of more than 2,500 meters, and are an important source of protein and fat for plateau herders, directly supporting the dietary nutritional needs of local residents, especially in remote pastoral areas, where meat and dairy products are often irreplaceable basic food. In order to adapt to extreme ecological environments such as alpine, hypoxia, and strong ultraviolet rays in plateau areas, Tibetan sheep have evolved a series of unique physiological characteristics and adaptive mechanisms (Sha et al., 2023). Due to natural grazing all year round, natural pasture becomes its main source of nutrients (An et al., 2005), and the quality of forage grass directly affects their growth and productivity (Piña et al., 2020). To maintain their growth, development and metabolism under extreme environmental conditions, the powerful rumen fermentation function provides them with the energy needed for survival and production (Jing et al., 2020).

The rumen is an important biological reaction vessel for ruminants, which contains a large number of microorganisms, and these bacteria, fungi, archaea, and protozoa together form a complex rumen microecosystem (Fan et al., 2020; Lin et al., 2019; Mizrahi et al., 2021). Microorganisms maintain nutrient absorption, metabolism and energy balance in the gastrointestinal tract by decomposing carbohydrates in forage grass to produce volatile fatty acids and ammonia nitrogen as energy and protein sources (Liu et al., 2019). Some difficult-to-digest carbohydrates are also produced as metabolites to provide energy after decomposition by intestinal flora (Louis and Flint, 2017). In addition, the yield and nutrient composition of natural grassland forages vary with altitude (Khiaosa-Ard et al., 2012). Changes in these factors lead to corresponding changes in the structure and metabolic function of the rumen flora (Ma et al., 2019; Zhang et al., 2016), however, it has not been clearly established how the composition and function of the rumen microflora of Tibetan sheep respond to changes in environmental factors. We hypothesized that the abundance of certain specific microbial taxa (e.g., hypoxia-tolerant or cold-tolerant bacteria) will increase significantly with altitude, and that certain metabolites associated with energy metabolism or immune regulation (e.g., volatile fatty acids, amino acid derivatives, etc.) will be significantly up-regulated. These flora and metabolites may play an important role in acclimatization to high altitude environments.

To validate this idea, we combined multi-omics techniques to analyze the changes of microbial communities and metabolites in Tibetan sheep adapted to different altitudes, and used amplicon sequencing and untargeted metabolism techniques to explore the distribution patterns and functional differences of some specific bacteria, fungi and metabolites in their bodies at different altitudes. We further integrate multi-omics data to deeply analyze the potential interactions between key microbial and metabolites, and look forward to providing new insights and theoretical basis for in-depth study on the mechanisms of gastrointestinal microbial-host-metabolite interactions and plateau adaptability of Tibetan sheep.

## 2 Materials and methods

### 2.1 Experimental animals and pastures

**Figure 1:**
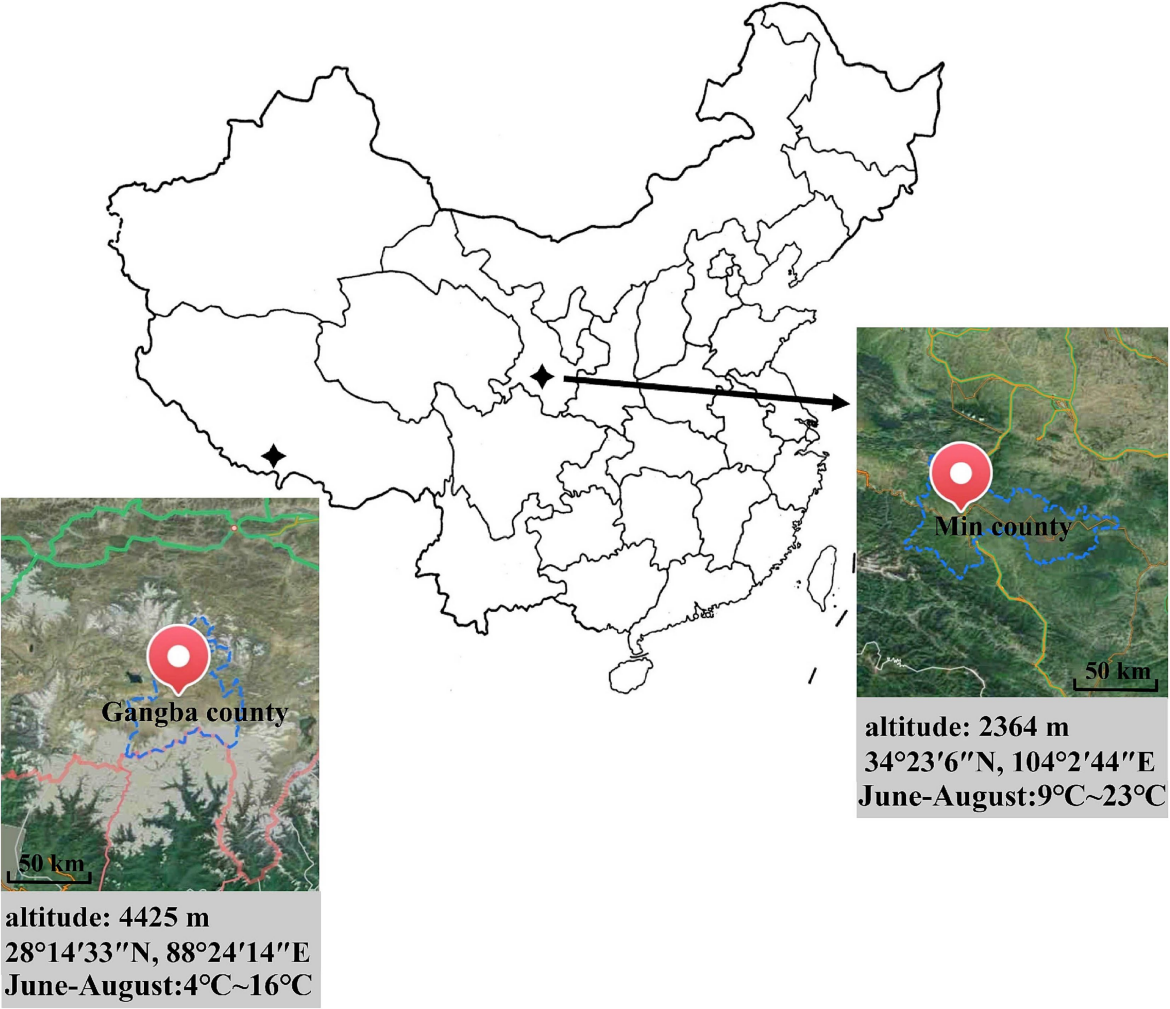
Geographic distribution map of Tibetan sheep.

Tibetan sheep freely grazing at two different altitudes were selected as study subjects (♂, weighing about 30.0 kg, *n* = 8/group), and all experimental animals were in good health. During the same period, they foraged freely on the mountain slopes without additional feed supplementation. The experiments were conducted in late August 2023 in Mende Village, Gangba County, Tibet Autonomous Region (28°14′33″N, 88°24′14″E, 4425 m) and Zana Village, Min County, Gansu Province (34°23′6″N, 104°2′44″E, 2364 m) (Figure 1). In this paper, G denotes the high altitude group and H denotes the low altitude group. The forage grass types in Gangba County are mainly alpine meadows, and the forage grasses are mostly Carex parvula O. Yano and Carex alatauensis S. R. Zhang (genus Carex, family Salix), which are highly regenerative, trample-resistant and resistant to grazing, while the forage types in Min County are mainly subalpine meadows and mountain meadows, and the sheep mostly feed on the palatable Leymus chinensis ((Trin.) Tzvel) (genus Lysimachia, family Gramineae).

### 2.2 Sample collection and measurement

A gastric tube rumen fluid sampler (MDW-15, Silicon Power, Shanghai, China) was used to collect rumen fluid from the oral cavity of each sheep before morning feeding. To avoid saliva contamination, 20 mL of rumen fluid was initially collected and discarded, and then 30 mL was collected and filtered through 4 layers of sterile gauze. Then, it was divided into freezing tubes and stored in liquid nitrogen for the determination of ammonium nitrogen (NH_3_-N), volatile fatty acids (VFAs), rumen microorganisms and metabolomics in rumen fluid. The NH_3_-N content was determined by the phenol-sodium hypochlorite colorimetric method on an enzyme labeling instrument (Thermo Electron Varioskan Flas, Shanghai Fuze Trading Co., Ltd.) (Broderick and Kang, 1980), and the VFAs were determined by Agilent Gas Chromatography (Agilent Technologies, Inc., Santa Clara, CA, United States) according to the method of Zheng et al. (2018). When collecting fecal samples, the sheep anus was stimulated with disposable sterile gloves to induce fecal discharge, and then the samples were quickly loaded into sterile cryopreservation tubes and immediately placed in liquid nitrogen for freezing, which was used for fecal microbiology and metabolomics testing.

### 2.3 Microbial amplicon sequencing and analysis

Rumen and fecal microbial DNA was extracted according to the instructions of the DNA extraction kit (Bio-Tek), and the concentration and purity were determined using a NanoDrop 2000 spectrophotometer (Thermo Fisher Scientific). Amplification of the V3-V4 variable region of the bacterial 16S rRNA gene was performed using primers 338F (5′-ACTCCTACGGGAGGCAGCAG-3′) and 806R (5′-GGACTACHVGGGTWTCTAAT-3′). Fungal ITS was amplified using ITS1F (5′-CTTGGTCATTTAGAGGAAGTAA-3′) and ITS2R (5′-GCTGCGTTCTTCATCGATGC-3′). After the preliminary experiment was completed, the formal PCR test was performed using a 20 μL reaction system (TransGen AP221-02: Trans Start Fastpfu DNA Polymerase and Pro Taq kit), with 3 replicates per sample. And the PCR products of the same samples were mixed and detected by electrophoresis on a 2.0% agarose gel, using the AxyPrepDNA Gel Recovery Kit (Axygen Biosciences, Silicon Valley, United States) to recover PCR products and purify them. Referring to the preliminary quantitative results of electrophoresis, the PCR products were detected and quantified using QuantiFluor™ -ST blue fluorescence quantification system and sequenced using Illumina Miseq PE300 platform by Shanghai Meiji Bio-pharmaceutical Technology Co.

High-quality raw data were spliced, filtered, chimera removal and noise reduction using fastp (v0.23.4) and FLASH software (version 1.2.11) to obtain valid data. Next the optimized sequences were clustered into operational taxonomic units (OTUs) using UPARSE 7.1 with 97% sequence similarity level. The taxonomy of each OTU representative sequence was analyzed by RDP Classifier (version 2.2) against the 16S rRNA gene database (e.g., Silva v138) using confidence threshold of 0.7. Then use R (version 3.3. 1) generate a Venn diagram to visualize the shared and unique OTUs between the two groups. Alpha diversity was used to assess microbial community diversity and species abundance, index analysis was performed using mothur (version v.1.30.2, University of Michigan, United States) and then *t*-test was used to test for differences between groups. PCoA analysis based on the Bray-Curtis distance was used to determine microbial β-diversity and to assess the similarity or difference in overall community structure between groups. Group differences were tested using Analysis of Similarities (ANOSIM) in R (version 3.3.1; vegan package). Origin 2021 v.9.8.0 (OriginLab, Northampton, MA, United States) and GraphPad Prism 9.1.2 (GraphPad Software, Te North Parker, United States) were used to process and visualize the microbial data. Finally, stamp software was used to analyze the microorganisms with significant differences between the two groups. And PICRUSt2 was used to predict KEGG function of rumen bacterial communities.

### 2.4 Untargeted metabolomics detection and analysis

Liquid chromatography-mass spectrometry was used for untargeted metabolomics determination of rumen fluid and feces. First, the sample metabolites were extracted. After slowly thawing rumen fluid and fecal samples on ice, 100 μL of rumen fluid and 50 mg of fecal samples were measured and placed into a 2-mL sterile centrifuge tube for operation, respectively. Add pre-cooled methanol/acetonitrile/water mixture (ratio of 2:2:1) to it, vortex mixed well, grind it at 50 Hz for 3 min, then ultrasonically crush at low temperature for 30 min, and then leave to stand at −20°C for 30 min. Centrifuge at 4°C for 15 min (15,294 × g, Rotor model: FA-45-30-11, Eppendorf 5430/R), and remove the supernatant for vacuum drying. Subsequently, 100 μL of compound solution (acetonitrile: water = 1:1, v/v) was added for compounding, vortexed, and centrifuged again for 10 min (4°C, 15294 × g), and the supernatant was pipetted into the injection vial for mass spectrometry analysis (Wang et al., 2019). In addition, 10 μL of supernatant was taken separately for each sample and mixed as a quality control sample.

Electrospray ionization of positive and negative modes was used for detection, respectively. The raw data were converted to mzXML format by ProteoWizard, and then the XCMS program was used to perform peak alignment, retention time correction, and extraction of the Peak areas were extracted using the XCMS program. Then the MS2 database was used for metabolite identification, and the data were preprocessed by the Ropls package in R language and then statistically analyzed. *T*-test and Principal Component Analysis (PCA) were used to examine the distribution of the samples between the two groups. Meanwhile, in order to overcome the shortcomings of PCA analysis that is insensitive to variables with small correlations, the metabolic profiles were further analyzed by orthogonal partial least squares-discriminant approach (Orthogonal PLS-DA, OPLS-DA), and the metrics of the OPLS-DA model, R_2_Y and Q_2_, were used to evaluate the model fitting ability and predictive ability, respectively, using the permutation test to assess the validity of the model estimation. In order to determine the metabolites and enrichment pathways that affect the significant difference between high and low-altitude Tibetan sheep, the variable importance for the projection (VIP) was obtained by the OPLS-DA model. The pretreated data were analyzed using a *t*-test and multiple difference analysis to obtain *p* values and FC values (Fold change). Different metabolites were screened using *p* < 0.05, FC > 2 as significant levels, and VIP > 1.0 as standard. Finally, the KEGG^1^ database and MetaboAnalyst 6.0 software was used to analyze potential biomarkers, and the significance level of metabolite enrichment in each pathway was analyzed and calculated through Fisher’s Exact Test, thereby determining the metabolic and signal transduction pathways that were significantly affected.

### 2.5 Statistical analysis

The rumen fluid fermentation parameters were assessed using *t*-test for multiple comparisons through IBM SPSS Statistics 27.0 software, and the results were expressed as mean and SEM. To control the false discovery rate (FDR), *p*-values from rumen fluid fermentation parameters, alpha diversity, stamp and KEGG analyses were adjusted using the Benjamini-Hochberg (BH) procedure. A corrected *p* < 0.05 was considered statistically significant. Spearman correlation analysis with FPC permutation was applied to examine microbe-metabolite relationships, where *p* < 0.05 were deemed statistically significant.

## 3 Results

### 3.1 Rumen fluid fermentation parameters

Comparison of rumen fluid fermentation parameters in Tibetan sheep at high and low altitudes showed that, after correction for FDR, NH_3_-N between group G and H remained significant (raw *p* < 0.001, *P*_FDR_ = 0.009), whereas the differences in the molar ratios of acetate and propionate and acetate -to-propionate ratio between the two groups were no longer significant (raw *p* = 0.027, *P*_FDR_ = 0.104; raw *p* = 0.046, *P*_FDR_ = 0.742; raw *p* = 0.012, *P*_FDR_ = 0.054) (Table 1).

**Table 1:** Fermentation parameters of rumen fluid in Tibetan sheep at different altitudes.

Items	LG	LH	SEM	*P*-value	P_FDR_-value
NH_3_-N, mg/L	134.57	97.86	5.972	<0.001	0.009
Total volatile fatty acids, mmol/L	101.57	94.92	9.904	0.742	0.081
Acetate, %	67.96	77.87	2.316	0.027	0.104
Propionate, %	17.94	12.37	1.425	0.046	0.742
Isobutyrate, %	1.85	0.84	0.420	0.245	0.315
Butyrate, %	9.53	7.36	0.576	0.056	0.084
Isovalerate, %	1.95	1.02	0.245	0.054	0.097
Valerate, %	0.76	0.54	0.157	0.511	0.575
Acetate/Propionate, A/P	4.61	6.49	0.397	0.012	0.054

LG, Rumen fluid of Tibetan sheep at high altitude. LH, Rumen fluid of Tibetan sheep at low altitude.

SEM, standard error of the mean.

Significant differences between the two groups are indicated by *p* < 0.05.

### 3.2 Comparison of microbial diversity in rumen fluid of Tibetan sheep at high and low altitudes

**Figure 2:**
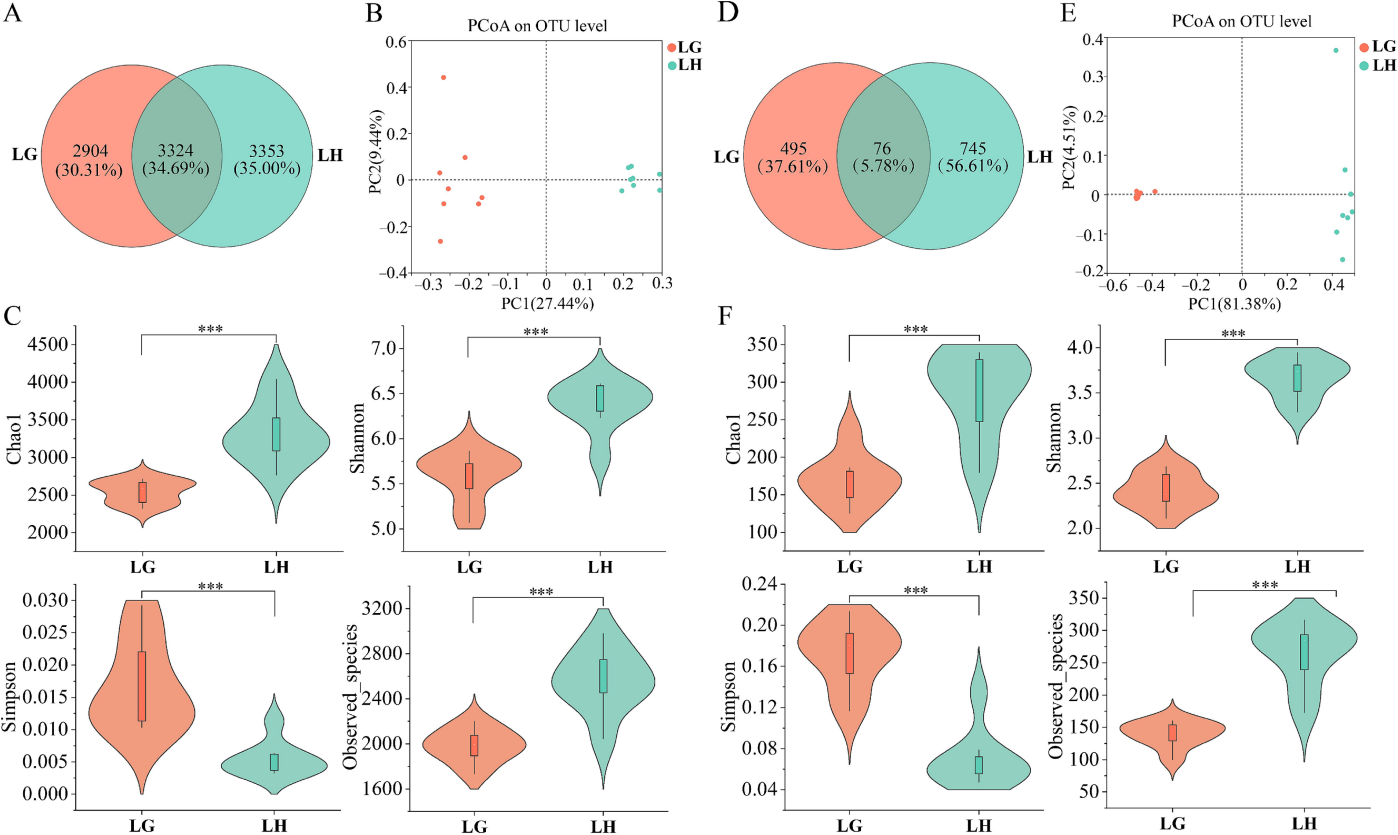
Microbial diversity in rumen fluid of Tibetan sheep at different altitudes. The Venn diagram illustrated the number of shared and unique OTUs in rumen bacterial **(A)** and fungal **(D)** communities between the two altitudes. PCoA plots highlight altitude-driven microbial divergence, **(B)** bacterial and **(E)** fungal. Estimation of bacterial **(C)** and fungal **(F)** community richness and diversity index. LG, Rumen fluid of Tibetan sheep at high altitude. LH, Rumen fluid of Tibetan sheep at low altitude. ***p < 0.001.

PCoA plots based on the Bray-Curtis distance metric showed differences in microbial diversity in Tibetan sheep between the two altitudes (Figures 2B,E). Alpha diversity (Figures 2C,F) showed that the results of bacterial and fungal diversity in rumen fluid were similar, and all indices showed extremely significant differences between the two groups, with chao1 index, Shannon index, and Observed_species significantly higher in group H than in group G, and the Simpson index was significantly lower than that in group G (*p* < 0.05). The number of OTUs unique to group H was also greater than that of group G in the bacterial and fungal communities (Figures 2A,D). It indicated that the diversity and richness of microbial community in the rumen fluid of Tibetan sheep in group H were higher than that in group G.

**Figure 3:**
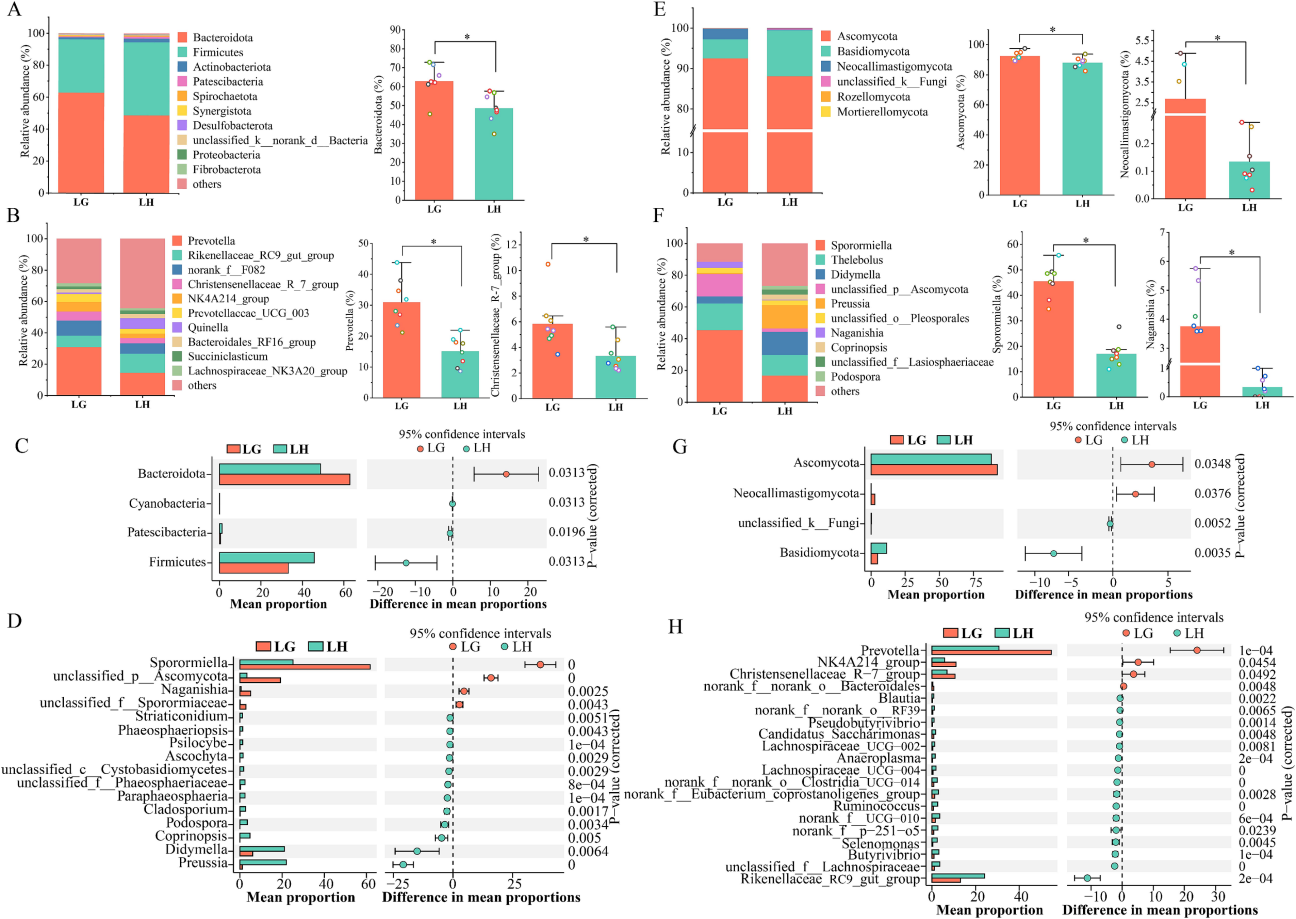
Comparison of rumen microorganisms in Tibetan sheep at different altitudes. The relative abundance of the top 10 **(A)** phylum and **(B)** genus of bacteria. Bacterial differential phylum **(C)** and differential genus **(D)**. The relative abundance of the top 10 **(E)** phylum and **(F)** genus of Fungi. Fungal differential phylum **(G)** and differential genus **(H)**. LG, Rumen fluid of Tibetan sheep at high altitude. LH, Rumen fluid of Tibetan sheep at low altitude.

Comparative analysis of bacteria in the rumen fluid of Tibetan sheep at high and low altitudes showed that a total of 4 significantly different phyla were identified at the phylum level (Figure 3C). Three phyla in group H were significantly higher in abundance than those in group G (*p* < 0.05). Among the top 10, the abundance of the Firmicutes, Patellobacteria, Desulfobacteria, and Proteobacteria were significantly higher than that of group G, while the abundance of Bacteroidota was significantly higher in group G (Figure 3A, *p* < 0.05). Figure 3D only showed the top 20 populations of different bacterial genera in the two groups, only 4 genera were significantly more abundant in group G than in group H, and the remaining 12 genera were significantly more abundant in group H (*p* < 0.05). However, among the top 10 populations at the genus level (Figure 3B; Supplementary Table S1), three populations of *Prevotella*, *Christensenellaceae R-7 group* and *NK4A214_group* were significantly higher in group G than in group H, and only *Rikenellaceae _ RC9 _ gut _ group* in group H was significantly higher than that in group G (*p* < 0.05).

**Figure 4:**
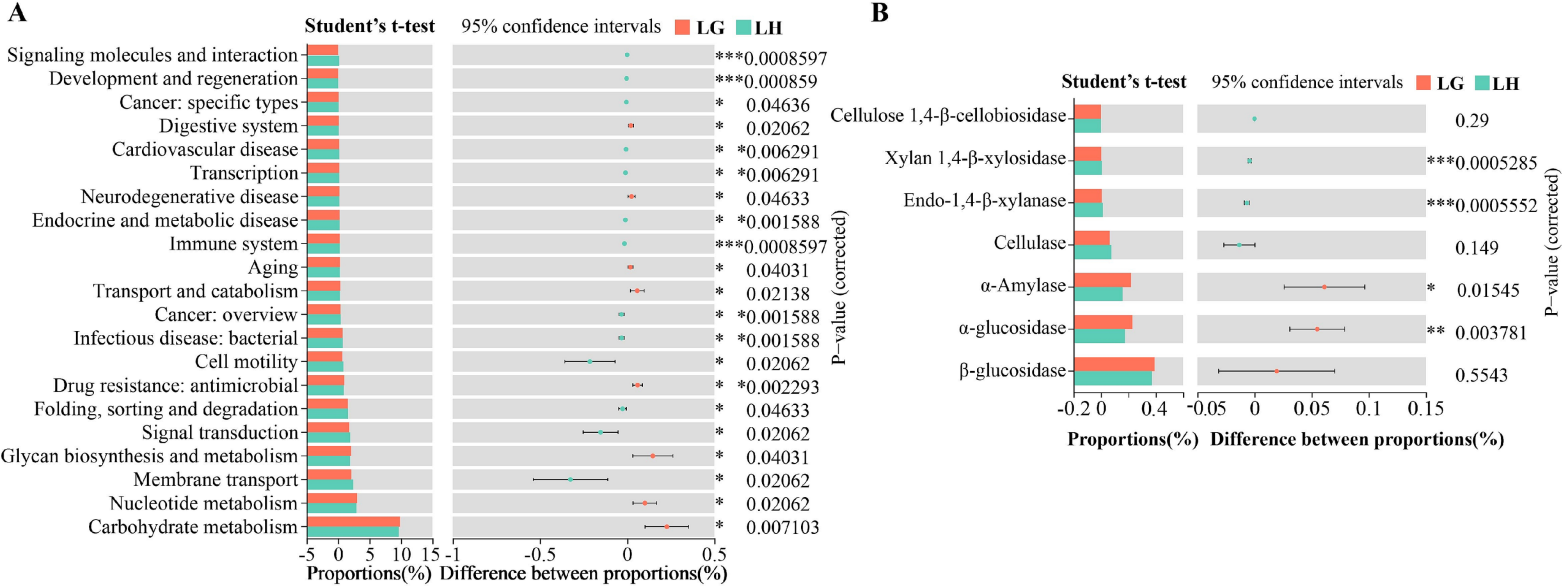
**(A)** KEGG function prediction analysis of rumen microbiota in Tibetan sheep at different altitudes. **(B)** Differential analysis of enzymes related to polysaccharide degradation in carbohydrate metabolic pathways. *p < 0.05, **p < 0.01, ***p < 0.001.

Analysis of fungi in rumen fluid showed that only 4 populations were identified at the phylum level, four of which were differential phylum (Figure 3G), of which the abundance of Ascomycota and Neocallimastigomycota in group G were significantly higher than that of group H. While the abundance of Basidiomycota and unclassified_k__Fungi had significantly lower abundance than group H (*p* < 0.05), and the remaining 2 populations were not significantly different (*p* > 0.05) (Figure 3E). At the genus level, Figure 3H showed the top 20 differential genera of fungi, with 4 genera significantly more abundant in group G than in group H, and the remaining 16 genera were significantly more abundant in group H than in group G (*p* < 0.05). Among the top 10 genera (Figure 3F; Supplementary Table S1), the abundance of *Sporormiella*, *unclassified _ p _ Ascomycota* and *Naganishia* were significantly higher in group G than in group H, the abundance of *Didymella*, *Preussia*, *Coprinopsis*, *unclassified _ f _ Lasiosphaeriaceae* and *Podospora* in group H were significantly more abundant than in group G (*p* < 0.05). Bacterial community KEGG function prediction plot showed 21 pathways that differed significantly between the two groups (Figure 4A). Carbohydrate metabolism function reached the highest and was significantly increased in group G (*p* < 0.05). Analysis of enzymes related to polysaccharide degradation in the Carbohydrate metabolism pathway revealed that the proportion of α-Amylase and α-glucosidase was significantly increased in group G (Figure 4B) (*p* < 0.05).

### 3.3 Comparison of microbial diversity in feces of Tibetan sheep at high and low altitudes

**Figure 5:**
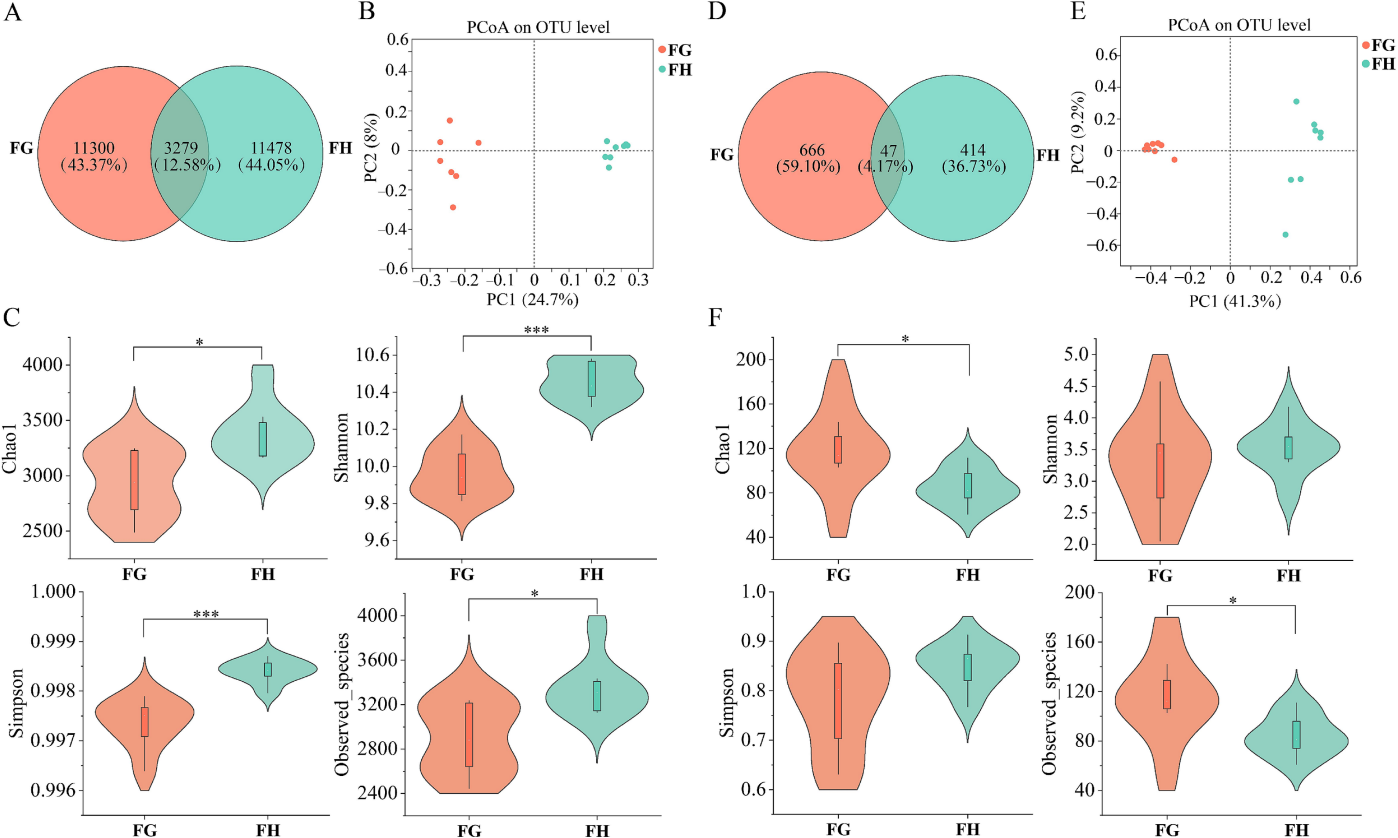
Microbial diversity of Tibetan sheep feces at different altitudes. The Venn diagram illustrated the number of shared and unique OTUs in feces bacterial **(A)** and fungal **(D)** communities between the two altitudes. PCoA plots highlight altitude-driven microbial divergence, **(B)** bacterial and **(E)** fungal. Estimation of bacterial **(C)** and fungal **(F)** community richness and diversity index. FG, Feces of Tibetan sheep at high altitude. FH, Feces of Tibetan sheep at low altitude. *p < 0.05, ***p < 0.001.

**Figure 6:**
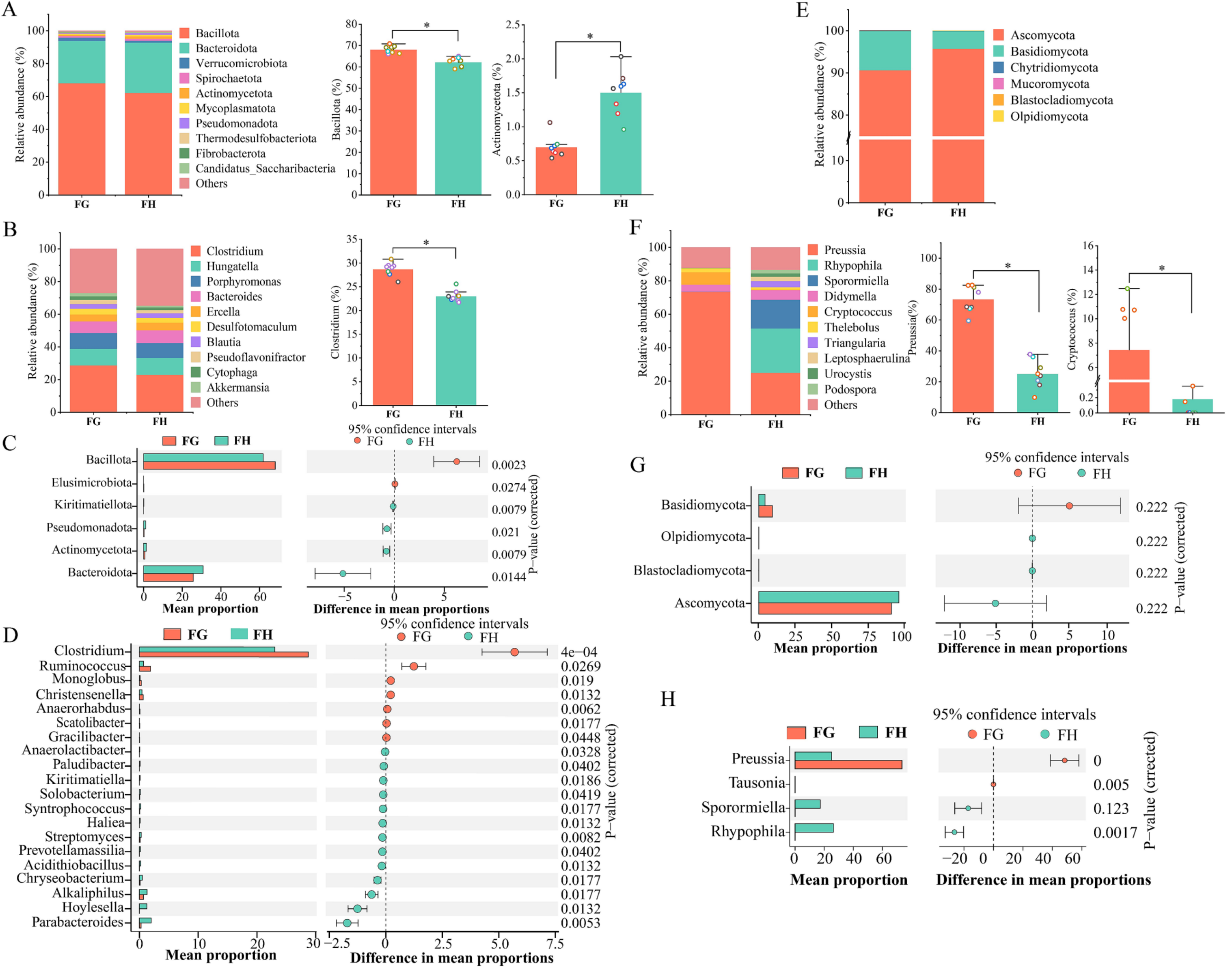
Comparison of fecal microorganisms in Tibetan sheep at different altitudes. The relative abundance of the top 10 **(A)** phylum and **(B)** genus of bacteria. Bacterial differential phylum **(C)** and differential genus **(D)**. The relative abundance of the top 10 **(E)** phylum and **(F)** genus of Fungi. Fungal differential phylum **(G)** and differential genus **(H)**. FG, Feces of Tibetan sheep at high altitude. FH, Feces of Tibetan sheep at low altitude.

At the same time, we also detected microbial diversity in Tibetan sheep feces. From the PCoA plot, it can be seen that the microbial diversity of Tibetan sheep feces varies greatly between the two altitudes (Figures 5B,E). Alpha diversity analysis of bacteria showed that Chao1, Shannon and Observed _ species in H group were significantly higher than those in G group, whereas these three indexes were significantly lower in fungi (Figures 5C,F). Venn analysis revealed a greater number of unique OTUs in group H compared to group G for rumen bacteria, while fungal communities showed an inverse distribution pattern (Figures 5A,D). The results showed that compared to high-altitude Tibetan sheep, low-altitude had higher diversity and richness (Chao 1) in fecal bacterial communities, while the Observe_species was less and the richness was lower in the fungal communities (*p* < 0.05). At the phylum level, a total of 6 differential bacterial populations were found between the two groups, in which the abundance of Bacillota and Elusimicrobiota were significantly higher in group G than in group H, and the abundance of the other 4 phyla in Group H were higher than that of group G (*p* < 0.05), namely Bacteroidota, Pseudomonadota, Actinomycetota, and Kiritimatiellota (Figures 6A,C; Supplementary Table S2). At the genus level, the top 20 significantly different genera were shown in Figure 6D, of which 13 genera had higher abundance in group H, and the remaining 7 genera were significantly higher in group G than in group H (*p* < 0.05). In the top 10, the abundance of all 9 genera in group G were higher than that in group H, with a significant increase in the abundance of *Clostridium* and *Desulfotomaculum* (*p* < 0.05) (Figure 6B; Supplementary Table S2). The fecal fungal community analysis between the two groups were as follows. A total of 6 fungal populations were identified at the phylum level, and none of these fungi were significantly different between the two groups (*p* > 0.05) (Figures 6E,G). At the genus level, there were 13 different genera (Figure 5H). Among the top 10 genera, the abundance of *Preussia* and *Cryptococcus* in group G were significantly higher than that in group H, while the *Rhypophila*, *Sporormiella*, and *Urocystis* were significantly lower than that in group H (*p* < 0.05) (Figure 6F; Supplementary Table S2).

### 3.4 Comparative analysis of rumen fluid metabolome of Tibetan sheep at high and low altitudes

**Figure 7:**
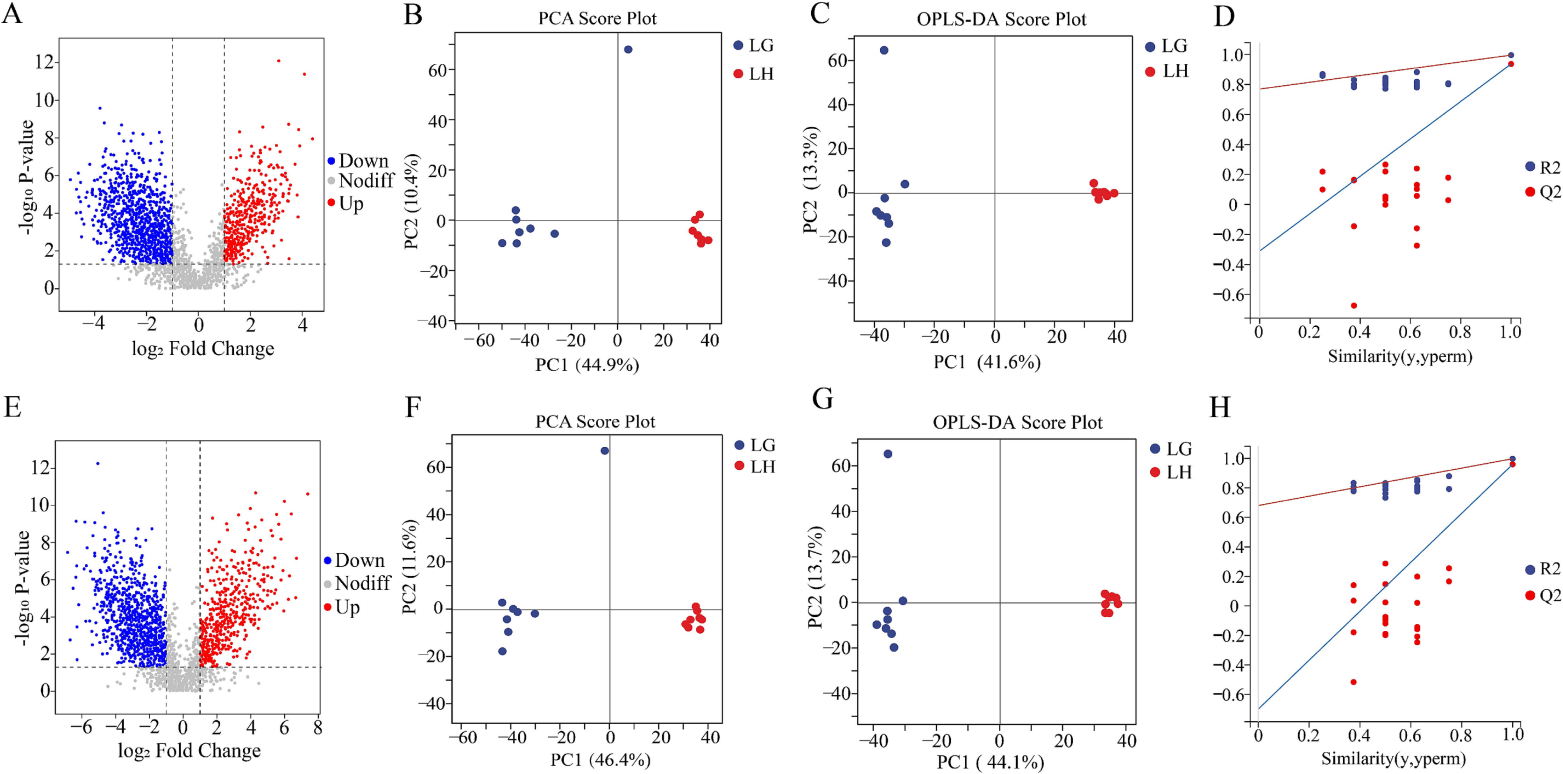
Comparison of rumen fluid metabolome of Tibetan sheep at different altitudes. Volcano plot **(A)**, Plot of PCA **(B)**, OPLS-DA score **(C)** and OPLS-DA permutation test **(D)** in the positive mode. Volcano plot **(E)**, Plot of PCA **(F)**, OPLS-DA score **(G)** and OPLS-DA permutation test **(H)** in the negative mode.

Differences of rumen microbial communities can cause changes in body metabolism. After studying the difference of rumen flora between high-altitude and low-altitude Tibetan sheep, in order to have a more in-depth understanding of the forage grass degradation function of the rumen flora as well as the differences in metabolites in the rumen fluid of Tibetan sheep, we used an untargeted metabolomics approach to study the metabolite. Metabolite profile volcano plots (Figures 7A,E) were plotted for statistical significance (FC > 2 or FC < 0.5, *p* < 0.05), with a total of 3,321 metabolites identified in the positive mode, and 2,965 metabolites identified in the negative mode. Overall, the number of metabolite species identified in the rumen fluid of Tibetan sheep in the two groups did not differ much, but relatively speaking, the number of up-regulated was higher in group G than in group H, which was related to the degradation function of its rumen flora.

**Figure 8:**
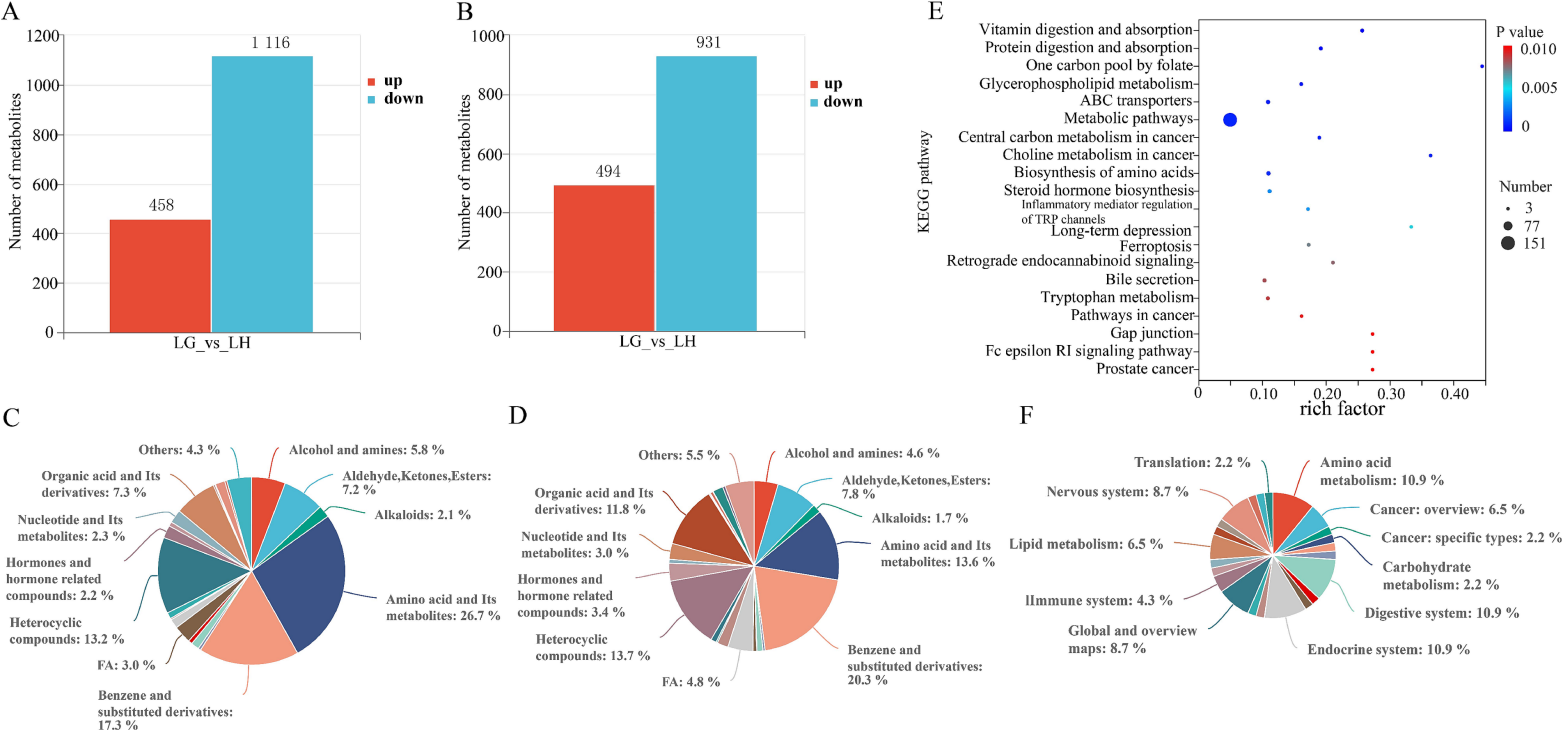
Differential metabolites and top 20 metabolic pathways in rumen fluid of Tibetan sheep at different altitudes. Differential metabolites **(A)** and compound classification statistics **(C)**, and KEGG pathway factor diagram **(E)** and its classification **(F)** in positive mode. Differential metabolites **(B)** and compound classification statistics **(D)** in negative mode.

The permutation test showed that all Q_2_ points are lower than the rightmost original Q_2_ point and the intersection of the regression line and the ordinate of the Q_2_ point is less than 0 (Figures 7D,H). Good separation of rumen metabolites was achieved between groups G and H as seen in the PCA and OPLS - DA plots in positive and negative modes (Figures 7B,C,F,G). The parameters used to assess the quality of the model for distinguishing between PCA and OPLS - DA of rumen fluid samples from groups G and H can be represented by the values in Supplementary Table S3 (R_2_X > 0.5, R_2_Y > 0.99). This indicates that the test results are reliable and valid, with significant differences in rumen metabolites of Tibetan sheep between the two altitudes. Subsequently, differential metabolite screening was carried out (VIP > 1.0, FC > 2.0 or FC < 0.5 and *p* < 0.05), and 1,574 differential metabolites were identified in positive mode, of which 458 metabolites were up-regulated in group H and 1,116 metabolites were up-regulated in group G. In the negative mode, a total of 1,425 differential metabolites were identified, of which 494 metabolites were up-regulated in group H and 931 metabolites were up-regulated in group G (Figures 8A,B). We classified the different metabolites in rumen fluid between the two groups, divided into 21 (positive) and 24 (negative) categories (Figures 8C,D). Overall, these metabolites account for the highest proportion among the four categories: Amino acid and Its metabolites, Benzene and substituted derivatives, Heterocyclic compounds, and Organic acid and Its derivatives.

Based on the screened differential metabolites, the pathway topology analysis was performed, and a total of 46 metabolic pathways had significant effects in the two groups (*p* < 0. 05) (Supplementary Table S4). The top 20 KEGG pathways with the most significant enrichment (FDR < 0.10, *p* < 0.01) were selected for display (Figure 8E), and the main enriched metabolic pathways included: Vitamin digestion and absorption, ABC transporters, Metabolic pathways, Biosynthesis of amino acids, Steroid hormone biosynthesis and Bile secretion. In addition, the 46 metabolic pathways with significant differences were classified and counted (level 2) (Figure 8F). The results showed that the main pathways affecting the metabolism of Tibetan sheep in the two groups were Amino acid metabolism, Digestive system, Endocrine system, Global and overview maps, Nervous system and Lipid metabolism. The differences in these metabolic pathways cause a series of responses in the body, which is one of the important mechanisms for the Tibetan sheep to adapt to the environment at this altitude.

### 3.5 Comparative analysis of fecal metabolome of Tibetan sheep at high and low altitudes

**Figure 9:**
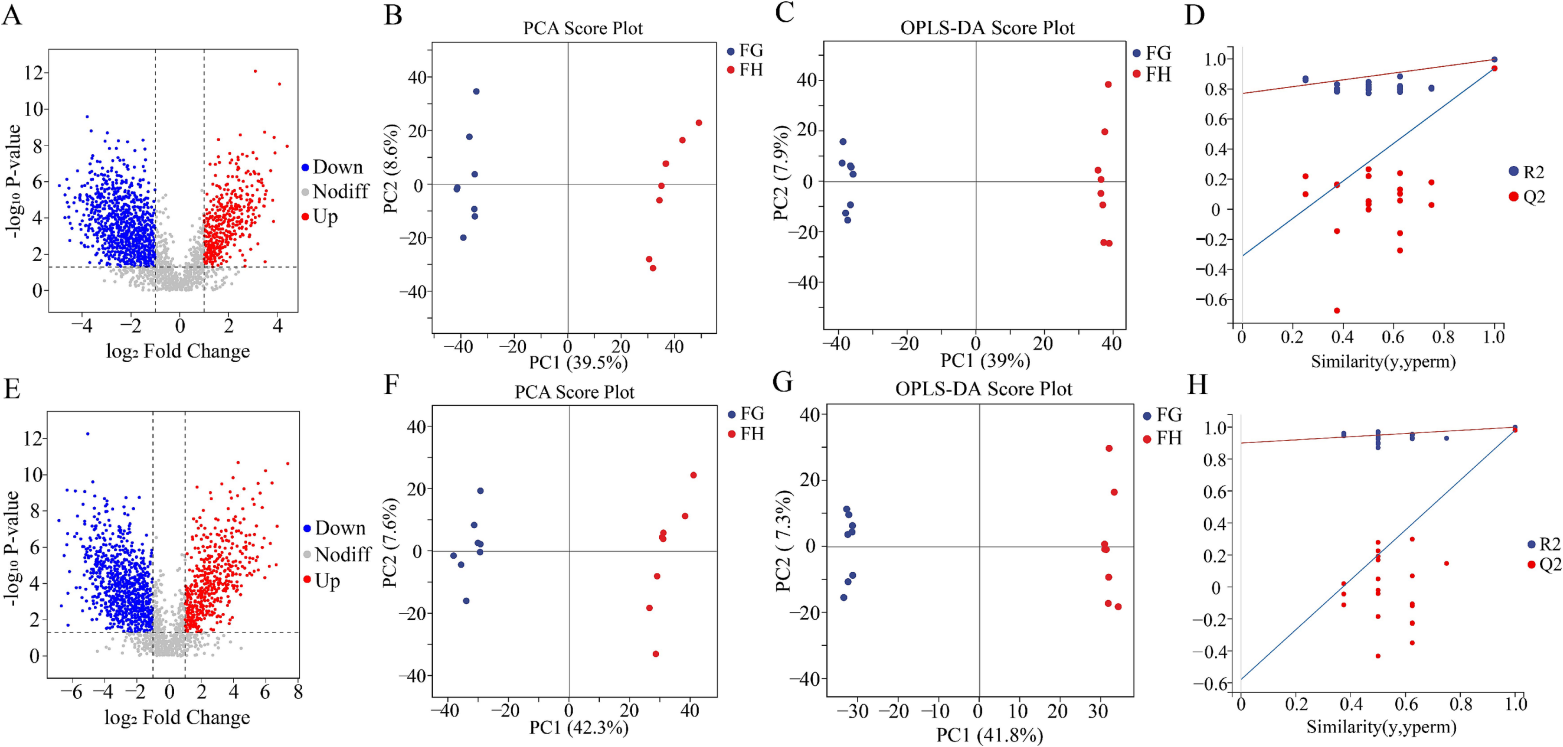
Comparison of fecal metabolome of Tibetan sheep at different altitudes. Volcano plot **(A)**, Plot of PCA **(B)**, OPLS-DA score **(C)** and OPLS-DA permutation test **(D)** in the positive mode. Volcano plot **(E)**, Plot of PCA **(F)**, OPLS-DA score **(G)** and OPLS-DA permutation test **(H)** in the negative mode.

Subsequently, to further explore the biological processes involved in the fecal excretion of differential metabolites from the rumen to the rectum, we also performed untargeted metabolite testing on rectal fecal samples from Tibetan sheep. Three thousand seven hundred and ninety metabolites were detected in positive mode and 2,645 in negative mode. From the analysis graphs of PCA and OPLS-DA, it can be seen that there are obvious divisions between the fecal metabolism groups between the two groups (Figures 9B,C,F,G), and it can be seen from the values of (R_2_X > 0.4, R_2_Y > 0.99) (Supplementary Table S3), and the prediction effect is relatively stable and reliable (Figures 9D,H). The volcano plots also showed a very clear distinction of differences, indicating that the metabolites are very different between the two groups (Figures 9A,E).

**Figure 10:**
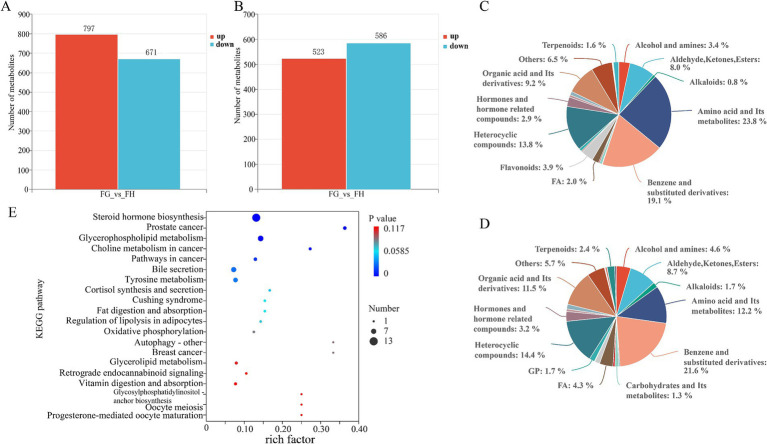
Differential metabolites and top 20 metabolic pathways in Tibetan sheep feces at different altitudes. Differential metabolites **(A)** and compound classification statistics **(C)**, and KEGG pathway factor diagram **(E)** in positive mode. Differential metabolites **(B)** and compound classification statistics **(D)** in negative mode.

Differential metabolites were screened according to the criteria of VIP > 1.0, FC > 2.0 or FC < 0.5 and *p* < 0.05. In the positive mode, there were 1,468 differential metabolites between the two groups, of which 797 metabolites were up-regulated in group H and 671 metabolites were up-regulated in group G. There were a total of 1,109 metabolites in negative mode, with 523 up-regulated in group H and 586 up-regulated in group G (Figures 10A,B). Classification of these differential metabolites revealed (Figures 10C,D) that, similar to the types of differential metabolites in rumen fluid, most of them were concentrated in the four categories of Amino acid and Its metabolites, Benzene and substituted derivatives, Heterocyclic compounds and Organic acid and Its derivatives. The KEGG database was used to analyze the fecal differential metabolites of Tibetan sheep in group G and group H, a total of 99 pathways were enriched. Figure 10E showed the top 20 KEGG pathways with the most significant enrichment, and only 7 pathways were significantly different (*p* < 0.05) (Supplementary Table S5). They are Steroid hormone biosynthesis, Prostate cancer, Glycerophospholipid metabolism, Choline metabolism in cancer, Pathways in cancer, Bile secretion and Tyrosine metabolism. Among them, Steroid hormone biosynthesis and Glycerophospholipid metabolism were classified as lipid metabolism. In these two pathways, there were 12 differential metabolites up-regulated in group H and 10 in group G, In Bile secretion, 4 differential metabolites were up-regulated in the H group and 3 in the G group, and 2 metabolites were up-regulated in the H group and 4 in the G group in Tyrosine metabolism.

### 3.6 Association analysis of differential microorganisms and differential metabolites

**Figure 11:**
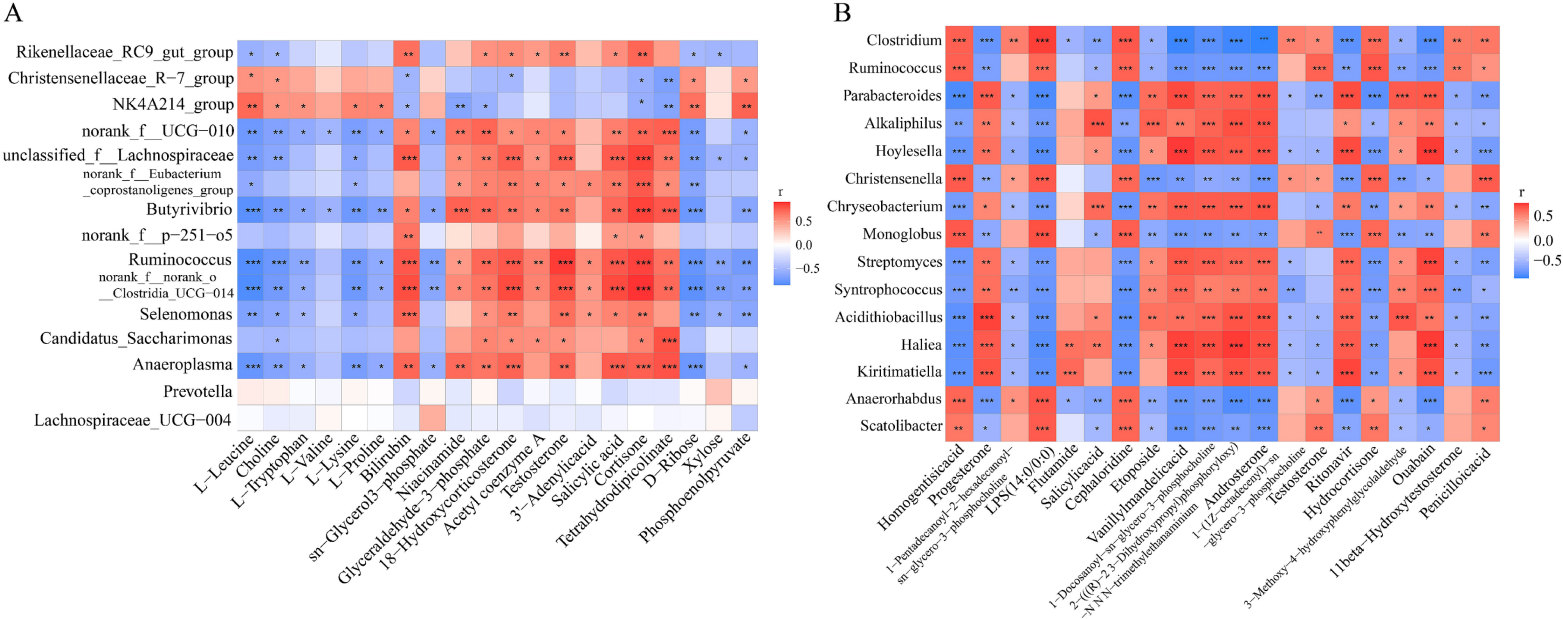
**(A)** Correlation analysis between differential bacteria in rumen fluid and main differential metabolites. **(B)** Correlation analysis between differential bacteria in feces and main differential metabolites. *p < 0.05, **p < 0.01, ***p < 0.001.

Differential bacterial genera of Tibetan sheep top15 were correlated with the major metabolites (Supplementary Table S6) enriched to the top20 KEGG pathway (positive) to tap into the link between the microorganisms and their metabolites. As shown in Figure 11A, a total of 98 positive and 73 negative correlations were found, of which *Butyrivibrio*, *Ruminococcus*, *Selenomonas* and *Anaeroplasma* were positively correlated with 7 metabolites, namely Bilirubin, Glyceraldehyde-3-phosphate, 18- Hydroxycorticosterone, Testosterone, Salicylic acid, Cortisone and Tetrahydrodipicolinate (*p* < 0.05). And negative correlation with all 6 metabolites of L-Leucine, Choline, L-Tryptophan, L-Lysine, D-Ribose and Phosphoenolpyruvate. *Christensenellaceae R-7 group* and *NK4A214_group* were positively correlated with the rumen metabolites L-Leucine, Choline, D-Ribose and Phosphoenolpyruvate (*p* < 0.05). *NK4A214_group* was also positively correlated with L-Tryptophan, L-Lysine and L-Proline (*p* < 0.05). *Butyrivibrio*, *Ruminococcus* and *Anaeroplasma* were positively correlated with Niacinamide and Tetrahydrodipicolinate. It was negatively correlated with L-Valine and sn-Glycerol 3-phosphate. *Butyrivibrio* and *Ruminococcus* were positively correlated with Acetyl coenzyme A (*p* < 0.05).

We also performed correlation analysis on the differential bacteria of feces and the metabolites (Supplementary Table S6) enriched in 7 differential pathways (Figure 11B), with a total of 130 positive correlations and 137 negative correlations. *Clostridium*, *Ruminococcus*, *Christensenella*, *Monoglobus*, *Anaerorhabdus* and *Scatolibacter* were positively correlated with Homogentisic acid, LPS (14:0/0:0), Cephaloridine, Testosterone, Hydrocortisone and Penicilloic acid (*p* < 0.05). With Progesterone, Etoposide, Vanillylmandelic acid, Androsterone, Ritonavir, and Ouabain G were negatively correlated (*p* < 0.05). While *Parabacteroides*, *Alkaliphilus*, *Hoylesella*, *Chryseobacterium*, *Streptomyces*, *Syntrophococcus*, *Acidithiobacillus*, *Haliea*, and *Kiritimatiella* were positively correlated with the above-mentioned 6 metabolites (except Etoposide) (*p* < 0.05). These 9 bacterias were negatively correlated with 6 metabolites of Homogentisic acid, LPS (14:0/0:0), Cephaloridine, Hydrocortisone, 11beta-Hydroxytestosterone and Penicilloic acid (*p* < 0.05).

## 4 Discussion

The forages ingested by ruminants are gradually decomposed into volatile fatty acids by the fermentation of various microorganisms in the rumen, providing more than 70% of the energy for the host body (Zanton and Heinrichs, 2009). Among them, acetate, propionate and butyrate is the main energy source of metabolic energy in ruminants (Jarrett and Filsell, 1961; Eskelandt et al., 1973), and propionate and butyrate can promote the development of rumen papilla (Lin et al., 2018). Tibetan sheep rely on natural forage grass to obtain nutrients for a long time, and the composition of forage grass is different due to the geographic location factors of the two regions. Compared with Leymus chinensis, Tibetan artemisia is high in crude protein, but after mid-August, the grass becomes harder, the lignin content (acid detergent fiber, Supplementary Table S7) increases, and the palatability decreases. The molar ratios of propionate and butyrate, NH_3_-N content in rumen fluid of Tibetan sheep in group G were significantly higher than those in group H, which was related to the forage grass intake and altitude. The decomposition of protein by rumen microorganisms will be enhanced by the higher content of crude protein in Artemisia grass. In this case, the high-abundance *Prevotella* are particularly active, making more NH_3_-N produced during forage fermentation (Assoumani et al., 1992; Zhou et al., 2018), which provides sufficient nitrogen source for microorganisms to synthesize their own cellular matter and promotes their growth and reproduction. At the same time, Bacteroidota are able to efficiently utilize nitrogen sources for rapid reproduction, thereby accelerating the degradation of high crude fibers in Artemisia grass and producing large amounts of butyrate and propionate, which further promotes the development of rumen papillae and enables Tibetan sheep to absorb nutrients more efficiently. Fan et al. (2020) also reported that in order to meet the energy demand in cold, high-altitude habitats, the rumen community of high-altitude yaks may have higher utilization capacity of high-fiber forage. From the perspective of energy utilization, acetate is mainly used to synthesize fats, while propionate provides energy to the host through the glycolenogenic pathway (Dijkstra, 1994; Bergman, 1990). High-altitude Tibetan sheep produce relatively more propionic acid in the rumen, but less acetic acid, resulting in a decrease in the A: P ratio. This implies that more energy is used for direct energy supply rather than fat synthesis. In order to ensure the survival in the environment of low oxygen, low temperature, etc., Tibetan sheep will preferentially use the acquired energy to maintain their own metabolic activities and physiological functions. The priority of energy distribution is one of the important strategies for Tibetan sheep to adapt to high altitude environment. Moreover, the microbial KEGG function prediction found that the high-altitude Tibetan sheep had richer Carbohydrate metabolism, Glycan biosynthesis and metabolism and Transport and catabolism functions. The above analyses indicated that high altitude Tibetan sheep have higher efficiency of carbohydrate catabolism, metabolism and energy utilization efficiency.

The diversity, community structure and metabolite types of rumen microorganisms are influenced by many factors such as host, diet composition and environmental conditions (Paz et al., 2016). Altitude affects the distribution, species and nutritional composition of forage, which in turn affects the diversity, composition and function of microorganisms in the rumen of Tibetan sheep (Wu et al., 2021). A large number of studies have shown that Firmicutes and Bacteroidota are always the dominant phylum in the rumen of ruminants such as cattle and sheep, although the proportion is slightly different (Andrade et al., 2020; Zhang et al., 2021; Ley et al., 2008; Myer et al., 2015). The composition of dominant bacteria in the rumen of Tibetan sheep in this study is similar to that. Bacteroidota can effectively decompose protein and carbohydrates in feed into VFAs, providing energy to the host (Jami et al., 2014) and promoting rumen development and volume increase (Lin et al., 2018). Importantly, in plateau hypoxia environments, VFAs can replace part of glucose metabolism and reduce oxygen-dependent energy consumption. While Firmicutes contribute to cellular uptake of glucose (Lin et al., 2018). The Bacteroidota in high altitude group increased significantly, indicating that Bacteroidota played a more important role in the adaptability of high altitudes environment than Firmicutes. In contrast, Huang et al. (2017) found that the relative abundance of the thick-walled phylum was higher than that of the anamorphic phylum in yaks during the grazing period. Due to the differences in living environments between the two regions, the changes in rumen microbial composition of grazing Tibetan sheep in this study were obvious. Significant differences in microbial community changes were also found in a comparative study of grazing yaks at three different altitudes (Xin et al., 2019). Fan et al. (2020) showed in their study of altitude affecting rumen microbial diversity in yaks that the increase in Christensenellaceae_R-7_group abundance with altitude was a good indication of the adaptation of yaks to the low temperature, low oxygen environment. *Christensenellaceae R-7 group* belong to a branch of the Firmicutes, and are involved in the decomposition of cellulose, starch and other compounds and are positively correlated with VFA production (Wei et al., 2022), but strangely, acetate is the opposite. It may be due to the fact that *Rikenellaceae_RC9_gut_group* are also involved in the degradation of cellulose (Qiu et al., 2020), with a significant increase in abundance in group H and a positive correlation with acetate (Wei et al., 2022). In addition, some fiber-degrading bacteria and sugar-fermenting bacteria (e.g., *Ruminococcus* and *Selenomonas*) produce hydrogen during fermentation, and the high abundance of these bacteria (in group H) directly increases the concentration of hydrogen in the rumen. Hydrogen will be used as a substrate for the acetogenic bacteria (*Blautia*) to produce more acetate. Although *Christensenellaceae R-7 group* are involved in in the degradation of cellulose, it is not clear which acid its metabolic pathway prefers to produce. These phenomena reflect the complexity of the rumen microbial community and the diversity of metabolic pathways, and the interactions between different bacterial communities together determines the proportion of final fermentation products.

Although rumen fungi represent only 5 to 20% of the overall rumen microflora, they also play an important function (Mani et al., 2022), with the ability to break down starch and hydrolyze protein activity (Guo et al., 2020). Compared to rumen bacteria, rumen fungi are more likely to degrade recalcitrant plant cell walls (Azad et al., 2020). Ascomycota and Basidiomycota are dominant fungi, which is similar to the rumen fungal composition of other ruminants such as cows (Yan et al., 2018). In this study, the abundance of Ascomycota, Neocallimastigomycota, *Sporormiella*, *unclassified _ p _ Ascomycota* and *Naganishia* in the rumen of Tibetan sheep in the high-altitude group was significantly increased. Neocallimastigomycota are the functional fungus commonly found in the digestive tract of herbivores and are considered to be the most effective bacteria to degrade lignin by producing ligninase (Gruninger et al., 2014). Both *Sporormiella* and *unclassified _ p _ Ascomycota* belong to Ascomycota. Ascomycota, as a class of higher fungi, are mainly involved in the degradation of organic substances such as lignin and keratin during nutrient digestion (Yan et al., 2018). *Naganishia* can survive in extreme conditions at high altitudes (Schmidt et al., 2017). These results further indicate that the composition of rumen microbial community has also undergone a series of changes in Tibetan sheep in order to adapt to the living conditions in high altitude areas. Unexpectedly, we only identified *Coprinopsis*, *unclassified_f__Lasiosphaeriaceae*, and *Podospora* in the rumen of low-altitude Tibetan sheep. The latter two all belong to the Ascomycota, commonly found in animal feces or decaying plants, and *Podospora* are also an important model for studying the mechanisms of autophagy and cell death (Pinan-Lucarré et al., 2007). *Coprinopsis* belong to Basidiomycota, which are also involved in the decomposition of complex carbohydrates such as cellulose, hemicellulose, etc. Some edible Basidiomycota containing polysaccharides can improve the body’s ability to inhibit tumors and rejection, and therefore become an important resource for the screening of antitumor drugs. Further studies are needed to gain a deeper understanding of the specific roles and effects of the Basidiomycota in the rumen of sheep.

Feces contain not only ration residues, products of metabolism, but also epithelial cells shed from the gastrointestinal mucosa and a large number of microorganisms. Since the fecal microbiota is related to gastrointestinal microorganisms (Olm and Sonnenburg, 2021), feces can also reflect, to some extent, the degree of feed utilization and the health of the gastrointestinal tract in ruminants. Similar to rumen microorganisms, based on the Alpha diversity analysis, there were more bacterial communities in the feces of Tibetan sheep at lower altitudes, but fewer fungal communities than at high altitude. Hoover (1978) showed that the hindgut segment of ruminants also plays an important role in the degradation of fibrous substances, and among the fecal bacteria, Bacillota (formerly known as Firmicutes) and Bacteroidota are still the dominant bacteria (Oikonomou et al., 2013; Huo et al., 2014). By annotating genes encoding carbohydrate enzymes, we learned that Bacillota, Bacteroidota, Pseudomonadota, and Kiritimatiellota have high cell wall degradation potential and play a key role in carbohydrate degradation and metabolism (Zhang et al., 2024). Pseudomonadota (Proteobacteria) are enriched in the gut and are susceptible to inflammation or invasion by exogenous pathogens, resulting in an imbalance in the microbial community (Auffret et al., 2017; Shin et al., 2015). Actinomycetota contain some bacteria that produce cellulases and antibacterial substances (Bull et al., 2005; Cundliffe, 2006), as well as bacteria that can affect intestinal health. Interestingly, in contrast to the rumen, the abundance of Bacillota were significantly higher and Bacteroidota were lower in group G. This may be related to the different forage grass species consumed by the two groups of sheep as well as the degree of re-catabolism and digestion in the digestive tract. The composition and function of gut microorganisms in animals living at high altitude are also related to host adaptation to harsh living conditions (Hao et al., 2024; Ma et al., 2019). Studies have reported that *Desulfotomaculum* can utilize H_2_ / CO_2_ autotrophic growth and produce sulfide or acetate (Aüllo et al., 2013). To a certain extent, the increase of this genus can compete with methanogens in the intestine for H_2_ and carbon sources, reducing the production of methane gas. This finding suggests that Tibetan sheep at high altitudes are more efficient in using energy. *Preussia* have also been shown to produce secondary metabolites with biological activity, especially antibacterial activity (Chen et al., 2009; Weber and Gloer, 1991). From the changes in the proportions of these flora compositions, it can be seen that the gut microbial community of Tibetan sheep at high altitude may help the host to adapt to the harsh survival environment by regulating energy metabolism and immune responses.

Microbial metabolites, as important mediators of microbial-host communication, mainly interact with the host by regulating glycolipid metabolic pathways and influencing the differentiation of immune cells to maintain host physiological homeostasis, such as bile acid, VFA, ammonia, phenol, and endotoxin (Wu et al., 2021). Microbial metabolites can promote microbial self-proliferation and interact with other metabolic factors to control microbial metabolism and related nutritional pathways (Bannink et al., 2016). At high altitudes, special conditions such as low oxygen levels may change the metabolic patterns of Tibetan sheep. Rumen metabolites are mostly enriched in amino acid metabolic pathways. Compared with low-altitude areas, Tibetan sheep at high altitude will have a special demand for certain essential amino acids, such as branched-chain amino acids, which may play an auxiliary role in preventing altitude sickness by regulating mitochondrial function and reducing the production of reactive oxygen species during the hypoxic stress response (Zandbergen et al., 2023). In contrast, at low altitudes, the amino acid metabolism in Tibetan sheep tends to favor a balance conducive to stable growth and reproductive states, such as the equilibrium regulation of muscle protein synthesis and degradation. Additionally, lipid metabolism is also more active (Conte et al., 2022), mainly involved in regulating the antibacterial effect of fatty acids and the hydrogenation of microorganisms (Jenkins, 1993). The correlation analysis between major differential metabolites and differential bacterial genera also found more correlations. In the rumen, *Christensenellaceae R-7 group* and *NK4A214 _ group* were positively correlated with metabolites L-Leucine, Choline, D-Ribose and Phosphoenolpyruvate with glycolysis and glyconeogenesis, and *NK4A214 _ group* were also positively correlated with L-Tryptophan, L-Lysine and L-Proline, while *Butyrivibrio*, *Ruminococcus*, *Selenomonas*, and *Anaeroplasma* were negatively correlated with the above six metabolites (except L-Proline). Amino acids are the basic units of peptides and proteins, which are activated to form proteins required for growth and development after a series of physiological and biochemical reactions in animals (Shi et al., 2023). L-Leucine, as a branched-chain fatty acid, has the effect of improving mitochondrial function and reducing oxidative stress. And Choline is a ‘lipophilic agent ‘that promotes the transport of fat in the form of phospholipids. D-ribose is a key factor involved in the biosynthesis of nucleotides, amino acids and cofactors with improved myocardial function (Akanmori et al., 2025). The abundances of *Christensenellaceae R-7 group* and *NK4A214_group* increased significantly at high altitudes, while the abundances of 4 populations such as *Butyrivibro* increased significantly at low altitudes. These changes in bacterial abundance and correlations with metabolites suggest that high-altitude Tibetan sheep may have a greater capacity for amino acid metabolism, lipid metabolism, glycolysis and gluconeogenesis, as well as improved myocardial function.

The metabolites in the 7 differential metabolic pathways in feces were mainly hormones and hormone-related compounds, glycerophospholipids and benzene and its substituted derivatives. We also found significant correlations in the association analysis of fecal differential bacteria and major metabolites. Five bacteria such as *Clostridium* were positively correlated with 6 metabolites of Homogentisic acid, LPS (14: 0 / 0: 0), Cephaloridine, Testosterone, Hydrocortisone and Penicilloic acid, and negative correlation was observed with metabolites such as Etoposide, Vanillylmandelic acid, Ritonavir and Ouabain G. While *Parabacteroides* and 9 other bacteria were positively correlated with Vanillylmandelic acid, Ritonavir and Ouabain G. Wang et al. (2008) have shown that *E. coli* in the intestine helps tyrosine to be converted into 4-hydroxyphenylpyruvic acid, which is then oxidized to produce Homogentisic acid. If some animals are congenitally deficient in Homogentisic acid oxidase, it will lead to Homogentisic acid cannot be degraded, disorder in the body’s metabolism, and ultimately cause homogenous acid disease. Testosterone can alter the structure of the intestinal microbiota (Jiang et al., 2023), causing changes in the permeability of the intestinal mucosal barrier, at which point the transfer of bacteria and their pro-inflammatory components to the body circulation, such as LPS produced by gut microorganisms, which may tilt the delicate balance toward tolerance or inflammation (Pither et al., 2024), resulting in a systemic low-level inflammatory response. This mechanism is a co-pathogenesis of diseases such as inflammatory bowel disease, irritable bowel syndrome and obesity (Bahlouli et al., 2020). It has been shown that commensal bacteria inhibit the growth of pathogens by controlling metabolic pathways of limited nutrient competition in the gut and by releasing antimicrobial substances (Wang and Kadarmideen, 2020). Vanillylmandelic acid is a major catecholamine metabolite used to diagnose tumor biomarkers (Baluchová et al., 2018). Cephaloridine (antibacterial), Hydrocortisone (anti-inflammatory), Penicilloic acid (antibacterial), Etoposide (anti-tumor), Ritonavir (antiviral), Ouabain G (sodium-potassium pump inhibitor, treatment of heart failure) are components of drugs used in the treatment of certain diseases and inhibit the growth of pathogens in the gut. Changes in intestinal metabolites are closely related to alterations in intestinal flora (Ottosson et al., 2018), and interactions between intestinal microorganisms and metabolites are an important part of maintaining homeostasis in the intestinal internal environment.

## 5 Conclusion

Joint microbiome and metabolome-based analysis reveals some key bacteria and metabolites for plateau adaptation in Tibetan sheep. Due to the survival environment at high altitudes, the ability of Tibetan sheep rumen microorganisms to ferment pasture grasses is enhanced (Bacteroidota), resulting in significantly higher molar ratios of propionate and butyrate and NH_3_-N content produced by fermentation, which provides sufficient nitrogen source and energy for rumen development and body metabolism. The rumen fungi Neocallimastigomycota and Ascomycota, which more readily degrade recalcitrant plant cell walls, have also responded positively. The abundance of Pseudomonadota, a phylum that tend to cause inflammation, and Actinomycetota, which contain cellulase-producing were significantly reduced in feces. In addition, differential bacteria in the rumen were mainly correlated with products related to amino acid and lipid metabolism, while differential bacterial genera in the feces were mainly correlated with a number of metabolites related to antimicrobial, anti-inflammatory, anti-tumor, and other drug components. These changes in microorganisms, metabolites, and microbial-metabolite interactions enable Tibetan sheep to better regulate the microecological balance of the gastrointestinal tract and the metabolism of the body to adapt to changes in the plateau environment.

## Data Availability

Raw 16S rRNA and ITS gene sequences from Tibetan sheep rumen fluid and fecal samples were in the NCBI Sequence Read Archive (SRA) repositories: PRJNA1235464 and PRJNA1235476. The raw rumen fluid and fecal metabolome data were deposited in MetaboLights with ID MTBLS12347 and MTBLS12351.

## Footnotes

^1^ http://www.genome.jp/kegg

## References

[ref1] Akanmori N. N. Junop M. S. Gupta R. S. Park J. (2025). Conformational flexibility of human ribokinase captured in seven crystal structures. Int. J. Biol. Macromol. 299:140109. doi: 10.1016/j.ijbiomac.2025.140109, PMID: 39837438

[ref2] An D. Dong X. Dong Z. (2005). Prokaryote diversity in the rumen of yak (*Bos grunniens*) and Jinnan cattle (*Bos taurus*) estimated by 16S rDNA homology analyses. Anaerobe 11, 207–215. doi: 10.1016/j.anaerobe.2005.02.001, PMID: 16701570

[ref3] Andrade B. G. Bressani F. A. Cuadrat R. R. Tizioto P. C. de Oliveira P. S. Mourão G. B.. (2020). The structure of microbial populations in Nelore GIT reveals inter-dependency of methanogens in feces and rumen. J. Anim. Sci. Biotechnol. 11, 1–10. doi: 10.1186/s40104-019-0422-x 32123563 PMC7038601

[ref4] Assoumani M. Vedeau F. Jacquot L. Sniffen C. (1992). Refinement of an enzymatic method for estimating the theoretical degradability of proteins in feedstuffs for ruminants. Anim. Feed Sci. Technol. 39, 357–368. doi: 10.1016/0377-8401(92)90054-A

[ref5] Auffret M. D. Dewhurst R. J. Duthie C.-A. Rooke J. A. John Wallace R. Freeman T. C.. (2017). The rumen microbiome as a reservoir of antimicrobial resistance and pathogenicity genes is directly affected by diet in beef cattle. Microbiome 5, 159–111. doi: 10.1186/s40168-017-0378-z, PMID: 29228991 PMC5725880

[ref6] Aüllo T. Ranchou-Peyruse A. Ollivier B. Magot M. (2013). Desulfotomaculum spp. and related gram-positive sulfate-reducing bacteria in deep subsurface environments. Front. Microbiol. 4:362. doi: 10.3389/fmicb.2013.00362, PMID: 24348471 PMC3844878

[ref7] Azad E. Fehr K. B. Derakhshani H. Forster R. Acharya S. Khafipour E.. (2020). Interrelationships of fiber-associated anaerobic fungi and bacterial communities in the rumen of bloated cattle grazing alfalfa. Microorganisms 8:1543. doi: 10.3390/microorganisms8101543, PMID: 33036363 PMC7601590

[ref8] Bahlouli W. Breton J. Lelouard M. L'Huillier C. Tirelle P. Salameh E.. (2020). Stress-induced intestinal barrier dysfunction is exacerbated during diet-induced obesity. J. Nutr. Biochem. 81:108382. doi: 10.1016/j.jnutbio.2020.108382, PMID: 32417626

[ref9] Baluchová S. Barek J. Tomé L. I. Brett C. M. Schwarzová-Pecková K. (2018). Vanillylmandelic and homovanillic acid: electroanalysis at non-modified and polymer-modified carbon-based electrodes. J. Electroanal. Chem. 821, 22–32. doi: 10.1016/j.jelechem.2018.03.011

[ref10] Bannink A. Van Lingen H. J. Ellis J. L. France J. Dijkstra J. (2016). The contribution of mathematical modeling to understanding dynamic aspects of rumen metabolism. Front. Microbiol. 7:1820. doi: 10.3389/fmicb.2016.01820, PMID: 27933039 PMC5120094

[ref11] Bergman E. (1990). Energy contributions of volatile fatty acids from the gastrointestinal tract in various species. Physiol. Rev. 70, 567–590. doi: 10.1152/physrev.1990.70.2.567, PMID: 2181501

[ref12] Broderick G. Kang J. (1980). Automated simultaneous determination of ammonia and total amino acids in ruminal fluid and *in vitro* media. J. Dairy Sci. 63, 64–75. doi: 10.3168/jds.s0022-0302(80)82888-8, PMID: 7372898

[ref13] Bull A. T. Stach J. E. Ward A. C. Goodfellow M. (2005). Marine actinobacteria: perspectives, challenges, future directions. Antonie Van Leeuwenhoek 87, 65–79. doi: 10.1007/s10482-004-6562-8, PMID: 15971359

[ref14] Chen X. Shi Q. Lin G. Guo S. Yang J. (2009). Spirobisnaphthalene analogues from the endophytic fungus Preussia sp. J. Nat. Prod. 72, 1712–1715. doi: 10.1021/np900302w, PMID: 19708679

[ref15] Conte G. Dimauro C. Daghio M. Serra A. Mannelli F. McAmmond B.. (2022). Exploring the relationship between bacterial genera and lipid metabolism in bovine rumen. Animal 16:100520. doi: 10.1016/j.animal.2022.100520, PMID: 35468508

[ref16] Cundliffe E. (2006). Antibiotic production by actinomycetes: the Janus faces of regulation. J. Ind. Microbiol. Biotechnol. 33:500. doi: 10.1007/s10295-006-0083-6, PMID: 16463161

[ref17] Dijkstra J. (1994). Production and absorption of volatile fatty acids in the rumen. Livest. Prod. Sci. 39, 61–69. doi: 10.1016/0301-6226(94)90154-6

[ref18] Eskelandt B. Pfander W. Preston R. (1973). Utilization of volatile fatty acids and glucose for protein deposition in lambs. Br. J. Nutr. 29, 347–355. doi: 10.1079/bjn19730113 4715147

[ref19] Fan Q. Wanapat M. Yan T. Hou F. (2020). Altitude influences microbial diversity and herbage fermentation in the rumen of yaks. BMC Microbiol. 20, 370–313. doi: 10.1186/s12866-020-02054-5, PMID: 33276718 PMC7718673

[ref20] Gruninger R. J. Puniya A. K. Callaghan T. M. Edwards J. E. Youssef N. Dagar S. S.. (2014). Anaerobic fungi (phylum Neocallimastigomycota): advances in understanding their taxonomy, life cycle, ecology, role and biotechnological potential. FEMS Microbiol. Ecol. 90, 1–17. doi: 10.1111/1574-6941.12383, PMID: 25046344

[ref21] Guo W. Wang W. Bi S. Long R. Ullah F. Shafiq M.. (2020). Characterization of anaerobic rumen fungal community composition in yak, Tibetan sheep and small tail han sheep grazing on the Qinghai-Tibetan plateau. Animals 10:144. doi: 10.3390/ani10010144, PMID: 31963125 PMC7023293

[ref22] Hao D. Niu H. Zhao Q. Shi J. An C. Wang S.. (2024). Impact of high-altitude acclimatization and de-acclimatization on the intestinal microbiota of rats in a natural high-altitude environment. Front. Microbiol. 15:1371247. doi: 10.3389/fmicb.2024.1371247, PMID: 38774503 PMC11106481

[ref23] Hoover W. H. (1978). Digestion and absorption in the hindgut of ruminants. J. Anim. Sci. 46, 1789–1799. doi: 10.2527/jas1978.4661789x, PMID: 567640

[ref24] Huang J. Li Y. Luo Y. (2017). Bacterial community in the rumen of Tibetan sheep and Gansu alpine fine-wool sheep grazing on the Qinghai-Tibetan plateau, China. J. Gen. Appl. Microbiol. 63, 122–130. doi: 10.2323/jgam.2016.08.003, PMID: 28239039

[ref25] Huo W. Zhu W. Mao S. (2014). Impact of subacute ruminal acidosis on the diversity of liquid and solid-associated bacteria in the rumen of goats. World J. Microbiol. Biotechnol. 30, 669–680. doi: 10.1007/s11274-013-1489-8, PMID: 24068532

[ref26] Jami E. White B. A. Mizrahi I. (2014). Potential role of the bovine rumen microbiome in modulating milk composition and feed efficiency. PLoS One 9:e85423. doi: 10.1371/journal.pone.0085423, PMID: 24465556 PMC3899005

[ref27] Jarrett I. Filsell O. (1961). An effect of glucose on acetate metabolism in sheep. Nature 190, 1114–1115. doi: 10.1038/1901114b0 13789588

[ref28] Jenkins T. (1993). Lipid metabolism in the rumen. J. Dairy Sci. 76, 3851–3863. doi: 10.3168/jds.s0022-0302(93)77727-9, PMID: 8132891

[ref29] Jiang X. Deng S. Lu N. Yao W. Xia D. Tu W.. (2023). Fecal microbial composition associated with testosterone in the development of Meishan male pigs. Front. Microbiol. 14:1257295. doi: 10.3389/fmicb.2023.1257295, PMID: 38053550 PMC10694212

[ref30] Jing X. Wang W. Degen A. Guo Y. Kang J. Liu P.. (2020). Tibetan sheep have a high capacity to absorb and to regulate metabolism of SCFA in the rumen epithelium to adapt to low energy intake. Br. J. Nutr. 123, 721–736. doi: 10.1017/s0007114519003222, PMID: 31813386

[ref31] Khiaosa-Ard R. Soliva C. Kreuzer M. Leiber F. (2012). Effects of species-diverse high-alpine forage on *in vitro* ruminal fermentation when used as donor cow's feed or directly incubated. Animal 6, 1764–1773. doi: 10.1017/s1751731112000717, PMID: 22717263

[ref32] Ley R. E. Hamady M. Lozupone C. Turnbaugh P. J. Ramey R. R. Bircher J. S.. (2008). Evolution of mammals and their gut microbes. Science 320, 1647–1651. doi: 10.1126/science.1155725, PMID: 18497261 PMC2649005

[ref33] Lin X. Wang J. Hou Q. Wang Y. Hu Z. Shi K.. (2018). Effect of hay supplementation timing on rumen microbiota in suckling calves. MicrobiologyOpen 7:e00430. doi: 10.1002/mbo3.430, PMID: 29280327 PMC5822350

[ref34] Lin L. Xie F. Sun D. Liu J. Zhu W. Mao S. (2019). Ruminal microbiome-host crosstalk stimulates the development of the ruminal epithelium in a lamb model. Microbiome 7, 83–16. doi: 10.1186/s40168-019-0701-y, PMID: 31159860 PMC6547527

[ref35] Liu C. Wu H. Liu S. Chai S. Meng Q. Zhou Z. (2019). Dynamic alterations in yak rumen bacteria community and metabolome characteristics in response to feed type. Front. Microbiol. 10:1116. doi: 10.3389/fmicb.2019.01116, PMID: 31191470 PMC6538947

[ref36] Louis P. Flint H. J. (2017). Formation of propionate and butyrate by the human colonic microbiota. Environ. Microbiol. 19, 29–41. doi: 10.1111/1462-2920.13589, PMID: 27928878

[ref37] Ma Y. Ma S. Chang L. Wang H. Ga Q. Ma L.. (2019). Gut microbiota adaptation to high altitude in indigenous animals. Biochem. Biophys. Res. Commun. 516, 120–126. doi: 10.1016/j.bbrc.2019.05.085, PMID: 31196622

[ref38] Ma L. Xu S. Liu H. Xu T. Hu L. Zhao N.. (2019). Yak rumen microbial diversity at different forage growth stages of an alpine meadow on the Qinghai-Tibet plateau. PeerJ 7:e7645. doi: 10.7717/peerj.7645, PMID: 31579584 PMC6754979

[ref39] Mani S. Aiyegoro O. A. Adeleke M. A. (2022). Association between host genetics of sheep and the rumen microbial composition. Trop. Anim. Health Prod. 54:109. doi: 10.1007/s11250-022-03057-2, PMID: 35192073

[ref40] Mizrahi I. Wallace R. J. Moraïs S. (2021). The rumen microbiome: balancing food security and environmental impacts. Nat. Rev. Microbiol. 19, 553–566. doi: 10.1038/s41579-021-00543-6, PMID: 33981031

[ref41] Myer P. R. Wells J. E. Smith T. P. Kuehn L. A. Freetly H. C. (2015). Microbial community profiles of the colon from steers differing in feed efficiency. Springerplus 4, 1–13. doi: 10.1186/s40064-015-1201-6 26322260 PMC4549364

[ref42] Oikonomou G. Teixeira A. G. V. Foditsch C. Bicalho M. L. Machado V. S. Bicalho R. C. (2013). Fecal microbial diversity in pre-weaned dairy calves as described by pyrosequencing of metagenomic 16S rDNA. Associations of *Faecalibacterium* species with health and growth. PLoS One 8:e63157. doi: 10.1371/journal.pone.0063157, PMID: 23646192 PMC3639981

[ref43] Olm M. R. Sonnenburg J. L. (2021). Ancient human faeces reveal gut microbes of the past. Nature 594, 182–183. doi: 10.1038/d41586-021-01266-7, PMID: 34007025

[ref44] Ottosson F. Brunkwall L. Ericson U. Nilsson P. M. Almgren P. Fernandez C.. (2018). Connection between BMI-related plasma metabolite profile and gut microbiota. J. Clin. Endocrinol. Metab. 103, 1491–1501. doi: 10.1210/jc.2017-02114, PMID: 29409054

[ref45] Paz H. A. Anderson C. L. Muller M. J. Kononoff P. J. Fernando S. C. (2016). Rumen bacterial community composition in Holstein and Jersey cows is different under same dietary condition and is not affected by sampling method. Front. Microbiol. 7:1206. doi: 10.3389/fmicb.2016.01206, PMID: 27536291 PMC4971436

[ref46] Piña L. F. Balocchi O. A. Keim J. P. Pulido R. G. Rosas F. (2020). Pre-grazing herbage mass affects grazing behavior, herbage disappearance, and the residual nutritive value of a pasture during the first grazing session. Animals 10:212. doi: 10.3390/ani10020212, PMID: 32012847 PMC7070351

[ref47] Pinan-Lucarré B. Paoletti M. Clavé C. (2007). Cell death by incompatibility in the fungus Podospora. Semin. Cancer Biol. 17, 101–111. doi: 10.1016/j.semcancer.2006.11.009, PMID: 17204431

[ref48] Pither M. D. Andretta E. Rocca G. Balzarini F. Matamoros-Recio A. Colicchio R.. (2024). Deciphering the chemical language of the immunomodulatory properties of *Veillonella parvula* lipopolysaccharide. Angew. Chem. Int. Ed. 63:e202401541. doi: 10.1002/anie.202401541, PMID: 38393988

[ref49] Qiu Q. Gao C. Ur Rahman M. A. Cao B. Su H. (2020). Digestive ability, physiological characteristics, and rumen bacterial community of Holstein finishing steers in response to three nutrient density diets as fattening phases advanced. Microorganisms 8:335. doi: 10.3390/microorganisms8030335, PMID: 32120877 PMC7142484

[ref50] Schmidt S. K. Vimercati L. Darcy J. L. Arán P. Gendron E. M. Solon A. J.. (2017). A *Naganishia* in high places: functioning populations or dormant cells from the atmosphere? Mycology 8, 153–163. doi: 10.1080/21501203.2017.1344154, PMID: 30123637 PMC6059072

[ref51] Sha Y. Guo X. He Y. Li W. Liu X. Zhao S.. (2023). Synergistic responses of Tibetan sheep rumen microbiota, metabolites, and the host to the plateau environment. Int. J. Mol. Sci. 24:14856. doi: 10.3390/ijms241914856, PMID: 37834304 PMC10573510

[ref52] Shi Z. Wang Y. Yan X. Ma X. Duan A. Hassan F.-u.. (2023). Metagenomic and metabolomic analyses reveal the role of gut microbiome-associated metabolites in diarrhea calves. mSystems 8:e0058223. doi: 10.1128/msystems.00582-23, PMID: 37615434 PMC10654109

[ref53] Shin N.-R. Whon T. W. Bae J.-W. (2015). Proteobacteria: microbial signature of dysbiosis in gut microbiota. Trends Biotechnol. 33, 496–503. doi: 10.1016/j.tibtech.2015.06.011, PMID: 26210164

[ref54] Wang X. Kadarmideen H. N. (2020). Metabolite genome-wide association study (mGWAS) and gene-metabolite interaction network analysis reveal potential biomarkers for feed efficiency in pigs. Meta 10:201. doi: 10.3390/metabo10050201, PMID: 32429265 PMC7281523

[ref55] Wang B. Ma M. P. Diao Q. Y. Tu Y. (2019). Saponin-induced shifts in the rumen microbiome and metabolome of young cattle. Front. Microbiol. 10:356. doi: 10.3389/fmicb.2019.00356, PMID: 30873143 PMC6403146

[ref56] Wang X. Villar V. A. M. Armando I. Eisner G. M. Felder R. A. Jose P. A. (2008). Dopamine, kidney, and hypertension: studies in dopamine receptor knockout mice. Pediatr. Nephrol. 23, 2131–2146. doi: 10.1007/s00467-008-0901-3, PMID: 18615257 PMC3724362

[ref57] Weber H. A. Gloer J. B. (1991). The preussomerins: novel antifungal metabolites from the coprophilous fungus *Preussia isomera* Cain. J. Org. Chem. 56, 4355–4360. doi: 10.1021/jo00014a007

[ref58] Wei X. Ouyang K. Long T. Liu Z. Li Y. Qiu Q. (2022). Dynamic variations in rumen fermentation characteristics and bacterial community composition during *in vitro* fermentation. Fermentation 8:276. doi: 10.3390/fermentation8060276

[ref59] Wu D. Vinitchaikul P. Deng M. Zhang G. Sun L. Wang H.. (2021). Exploration of the effects of altitude change on bacteria and fungi in the rumen of yak (*Bos grunniens*). Arch. Microbiol. 203, 835–846. doi: 10.1007/s00203-020-02072-x, PMID: 33070234

[ref60] Wu J. Wang K. Wang X. Pang Y. Jiang C. (2021). The role of the gut microbiome and its metabolites in metabolic diseases. Protein Cell 12, 360–373. doi: 10.1007/s13238-020-00814-7, PMID: 33346905 PMC8106557

[ref61] Xin J. Chai Z. Zhang C. Zhang Q. Zhu Y. Cao H.. (2019). Comparing the microbial community in four stomach of dairy cattle, yellow cattle and three yak herds in Qinghai-Tibetan plateau. Front. Microbiol. 10:1547. doi: 10.3389/fmicb.2019.01547, PMID: 31354656 PMC6636666

[ref62] Xu T. Zhao N. Hu L. Xu S. Liu H. Ma L.. (2017). Characterizing CH4, CO2 and N2O emission from barn feeding Tibetan sheep in Tibetan alpine pastoral area in cold season. Atmos. Environ. 157, 84–90. doi: 10.1016/j.atmosenv.2017.03.023

[ref63] Yan X. Yan B. Ren Q. Dou J. Wang W. Zhang J.. (2018). Effect of slow-release urea on the composition of ruminal bacteria and fungi communities in yak. Anim. Feed Sci. Technol. 244, 18–27. doi: 10.1016/j.anifeedsci.2018.07.016

[ref64] Zandbergen L. Van Dijk C. Sen P. Sorop O. Van Drie R. Shashikadze B.. (2023). Impaired cardiac BCAA catabolism associated with impaired myocardial efficiency during exercise in a porcine model with multiple risk factors. Eur. Heart J. 44:ehad655.3162. doi: 10.1093/eurheartj/ehad655.3162

[ref65] Zanton G. Heinrichs A. (2009). Digestion and nitrogen utilization in dairy heifers limit-fed a low or high forage ration at four levels of nitrogen intake. J. Dairy Sci. 92, 2078–2094. doi: 10.3168/jds.2008-1712, PMID: 19389966

[ref66] Zhang C. La Y. Ma X. Zhandui P. Wu X. Guo X.. (2024). The effects of different doses of compound enzyme preparations on the production performance, meat quality and rumen microorganisms of yak were studied by metagenomics and transcriptomics. Front. Microbiol. 15:1491551. doi: 10.3389/fmicb.2024.1491551, PMID: 39726957 PMC11670318

[ref67] Zhang Z. Xu D. Wang L. Hao J. Wang J. Zhou X.. (2016). Convergent evolution of rumen microbiomes in high-altitude mammals. Curr. Biol. 26, 1873–1879. doi: 10.1016/j.cub.2016.05.012, PMID: 27321997

[ref68] Zhang Y. Zhang X. Li F. Li C. Li G. Zhang D.. (2021). Characterization of the rumen microbiota and its relationship with residual feed intake in sheep. Animal 15:100161. doi: 10.1016/j.animal.2020.100161, PMID: 33785185

[ref69] Zheng C. Li F. Hao Z. Liu T. (2018). Effects of adding mannan oligosaccharides on digestibility and metabolism of nutrients, ruminal fermentation parameters, immunity, and antioxidant capacity of sheep. J. Anim. Sci. 96, 284–292. doi: 10.1093/jas/skx040, PMID: 29385475 PMC6140840

[ref70] Zhou J. Liu H. Zhong C. Degen A. Yang G. Zhang Y.. (2018). Apparent digestibility, rumen fermentation, digestive enzymes and urinary purine derivatives in yaks and Qaidam cattle offered forage-concentrate diets differing in nitrogen concentration. Livest. Sci. 208, 14–21. doi: 10.1016/j.livsci.2017.11.020

