# Supplementary material for: Multi-omics insights into key microorganisms and metabolites in Tibetan sheep’s high-altitude adaptation
Source: Front Microbiol. 2025 Jun 18;16:1616555. doi: 10.3389/fmicb.2025.1616555 (PMC12213699; doi:10.3389/fmicb.2025.1616555)
Supplement: Supplementary file 2 [file Supplementary_file_2.docx]

Supplementary Material

# Supplementary Tables

Table S1 Top 10 phyla and genera in relative abundance among rumen bacteria and fungi.

| **Items** | **LG** | **LH** | **SEM** | ***P*-value** |
| --- | --- | --- | --- | --- |
| **Phylum level of rumen bacteria** | | | | |
| Bacteroidota | 62.99 | 48.75 | 2.666 | 0.003 |
| Firmicutes | 33.28 | 45.73 | 2.45 | 0.006 |
| Actinobacteriota | 1.28 | 2.30 | 0.307 | 0.099 |
| Patescibacteria | 0.49 | 1.23 | 0.125 | <0.001 |
| Spirochaetota | 0.56 | 0.60 | 0.083 | 0.837 |
| Synergistota | 0.40 | 0.34 | 0.139 | 0.849 |
| Desulfobacterota | 0.25 | 0.38 | 0.034 | 0.048 |
| unclassified_k__norank_d__Bacteria | 0.35 | 0.21 | 0.047 | 0.151 |
| Proteobacteria | 0.08 | 0.14 | 0.013 | 0.017 |
| Fibrobacterota | 0.10 | 0.06 | 0.017 | 0.279 |
| Others | 0.23 | 0.26 | 0.024 | 0.542 |
| **Genus level of rumen bacteria** | | | | |
| Prevotella | 30.99 | 14.64 | 2.713 | <0.001 |
| Rikenellaceae_RC9_gut_group | 7.22 | 12.11 | 0.817 | <0.001 |
| norank_f__F082 | 9.66 | 6.61 | 1.227 | 0.229 |
| Christensenellaceae_R_7_group | 5.85 | 3.41 | 0.545 | 0.018 |
| NK4A214_group | 6.04 | 2.79 | 0.732 | 0.02 |
| Prevotellaceae_UCG_003 | 5.06 | 3.15 | 0.602 | 0.116 |
| Quinella | 1.00 | 6.69 | 1.483 | 0.051 |
| Bacteroidales_RF16_group | 2.13 | 2.71 | 0.341 | 0.416 |
| Succiniclasticum | 1.73 | 2.27 | 0.338 | 0.443 |
| Lachnospiraceae_NK3A20_group | 2.05 | 1.41 | 0.288 | 0.283 |
| Others | 28.26 | 44.20 | 2.254 | <0.001 |
| **Phylum level of ruminal Fungi** | | | | |
| Ascomycota | 92.50 | 88.10 | 0.97 | 0.017 |
| Basidiomycota | 4.71 | 11.37 | 1.118 | <0.001 |
| Neocallimastigomycota | 2.69 | 0.14 | 0.405 | 0.001 |
| unclassified_k__Fungi | 0.07 | 0.37 | 0.051 | 0.001 |
| Rozellomycota | 0.02 | 0.00 | 0.011 | 0.392 |
| Mortierellomycota | 0.00 | 0.02 | 0.006 | 0.172 |
| **Genus level of rumen fungi** | | | | |
| Sporormiella | 45.55 | 16.77 | 4.127 | <0.001 |
| Thelebolus | 16.72 | 13.03 | 1.184 | 0.124 |
| Didymella | 4.42 | 14.65 | 2.095 | 0.009 |
| unclassified_p__Ascomycota | 14.08 | 2.13 | 1.659 | <0.001 |
| Preussia | 0.78 | 14.66 | 2.025 | <0.001 |
| unclassified_o__Pleosporales | 3.19 | 3.00 | 0.253 | 0.727 |
| Naganishia | 3.76 | 0.36 | 0.503 | <0.001 |
| Coprinopsis | 0.00 | 3.26 | 0.549 | <0.001 |
| unclassified_f__Lasiosphaeriaceae | 0.00 | 3.16 | 0.699 | 0.017 |
| Podospora | 0.00 | 2.41 | 0.423 | 0.001 |
| Others | 11.50 | 26.57 | 2.554 | 0.001 |

LG, Rumen fluid of Tibetan sheep at high altitude. LH, Rumen fluid of Tibetan sheep at low altitude;

Significant differences between the two groups are indicated by *P* < 0.05;

SEM, standard error of the mean.

Table S2 Top 10 phyla and genera in relative abundance of fecal bacteria and fungi.

| **Items** | **FG** | **FH** | **SEM** | ***P*-value** |
| --- | --- | --- | --- | --- |
| **Phylum level of fecal bacteria** | | | | |
| Bacillota | 68.11 | 62.17 | 0.907 | <0.001 |
| Bacteroidota | 25.68 | 30.59 | 0.845 | <0.001 |
| Verrucomicrobiota | 2.07 | 1.15 | 0.373 | 0.227 |
| Spirochaetota | 0.97 | 1.26 | 0.112 | 0.205 |
| Actinomycetota | 0.70 | 1.50 | 0.121 | <0.001 |
| Mycoplasmatota | 0.68 | 0.83 | 0.044 | 0.099 |
| Pseudomonadota | 0.35 | 1.05 | 0.117 | <0.001 |
| Thermodesulfobacteriota | 0.39 | 0.41 | 0.035 | 0.752 |
| Fibrobacterota | 0.43 | 0.14 | 0.106 | 0.177 |
| Candidatus_Saccharibacteria | 0.20 | 0.32 | 0.031 | 0.057 |
| Others | 0.42 | 0.57 | 0.063 | 0.233 |
| **Genus level of fecal bacteria** | | | | |
| Clostridium | 1.45 | 1.23 | 0.804 | <0.001 |
| Hungatella | 2.25 | 1.39 | 0.452 | 0.899 |
| Porphyromonas | 0.80 | 1.43 | 0.287 | 0.404 |
| Bacteroides | 1.44 | 1.19 | 0.33 | 0.344 |
| Ercella | 0.56 | 0.31 | 0.122 | 0.096 |
| Desulfotomaculum | 0.84 | 0.29 | 0.176 | 0.042 |
| Blautia | 0.61 | 0.43 | 0.128 | 0.913 |
| Pseudoflavonifractor | 0.67 | 0.37 | 0.148 | 0.069 |
| Cytophaga | 0.88 | 0.41 | 0.184 | 0.092 |
| Akkermansia | 2.03 | 0.46 | 0.375 | 0.231 |
| Others | 1.92 | 2.57 | 1.143 | <0.001 |
| **Phylum level of fecal Fungi** | | | | |
| Ascomycota | 90.64 | 95.75 | 1.604 | 0.113 |
| Basidiomycota | 9.32 | 4.20 | 1.602 | 0.113 |
| Chytridiomycota | 0.01 | 0.02 | 0.007 | 0.505 |
| Mucoromycota | 0.03 | 0.00 | 0.014 | 0.318 |
| Blastocladiomycota | 0.00 | 0.02 | 0.005 | 0.078 |
| Olpidiomycota | 0.00 | 0.00 | 0.000 | 0.132 |
| **Genus level of fecal Fungi** | | | | |
| Preussia | 73.45 | 25.05 | 6.614 | <0.001 |
| Rhypophila | 0.01 | 26.43 | 3.641 | <0.001 |
| Sporormiella | 0.15 | 17.27 | 2.894 | <0.001 |
| Didymella | 4.11 | 5.75 | 1.200 | 0.514 |
| Cryptococcus | 7.44 | 0.18 | 1.152 | 0.001 |
| Thelebolus | 2.31 | 1.46 | 0.450 | 0.364 |
| Triangularia | 0.00 | 3.65 | 1.562 | 0.256 |
| Leptosphaerulina | 0.00 | 2.67 | 0.773 | 0.083 |
| Urocystis | 0.23 | 1.99 | 0.349 | 0.006 |
| Podospora | 0.00 | 2.14 | 0.746 | 0.157 |
| Others | 12.30 | 13.40 | 2.780 | 0.851 |

FG, Feces of Tibetan sheep at high altitude. FH, Feces of Tibetan sheep at low altitude;

Significant differences between the two groups are indicated by *P* < 0.05;

SEM, standard error of the mean.

Table S3 Summary of the mode fitness of UHPLC-QTOF/MS analysis about the comparison between rumen fluid and feces of Tibetan sheep at high altitude and low altitude.

| **Items** | **Ion mode** | **Type^a^** | **R_2_X(cum)** | **R_2_Y(cum)** | **Q_2_(cum)** |
| --- | --- | --- | --- | --- | --- |
| Rumen fluid | Positive | PCA-QC | 0.553 |  |  |
|  |  | OPLS-DA | 0.550 | 0.996 | 0.937 |
|  | Negative | PCA-QC | 0.580 |  |  |
|  |  | OPLS-DA | 0.578 | 0.997 | 0.959 |
| Feces | Positive | PCA-QC | 0.540 |  |  |
|  |  | OPLS-DA | 0.470 | 0.999 | 0.978 |
|  | Negative | PCA-QC | 0.552 |  |  |
|  |  | OPLS-DA | 0.491 | 0.999 | 0.980 |

Note: R_2_X-the interpretability of model. Generally, R_2_ above 0.5 is better.

Table S7 Nutrient composition of pasture grasses at different altitudes

| **Items** | **G** | **H** | **SEM** | ***P*-value** |
| --- | --- | --- | --- | --- |
| Dry matter, % | 91.56 | 91.01 | 0.773 | 0.730 |
| Neutral detergent fiber, % | 35.17 | 43.97 | 0.957 | <0.001 |
| Acid detergent fiber, % | 41.68 | 38.66 | 0.683 | 0.025 |
| Crude protein, % | 11.37 | 8.12 | 0.373 | <0.001 |

G, High altitude group; H, Low altitude group;

Significant differences between the two groups are indicated by *P* < 0.05;

SEM, standard error of the mean.
